# Population-Based Psychiatric Comorbidity in Children and Adolescents With Autism Spectrum Disorder: A Meta-Analysis

**DOI:** 10.3389/fpsyt.2022.856208

**Published:** 2022-05-23

**Authors:** Tuba Mutluer, Herdem Aslan Genç, Aslihan Özcan Morey, Hale Yapici Eser, Beliz Ertinmaz, Merve Can, Kerim Munir

**Affiliations:** ^1^Department of Psychiatry, School of Medicine, Koç University, Istanbul, Turkey; ^2^Research Center for Translational Medicine (KUTTAM), Koç University, Istanbul, Turkey; ^3^Division of Child and Adolescent Psychiatry, Koç University Hospital, Istanbul, Turkey; ^4^School of Medicine, Koç University, Istanbul, Turkey; ^5^Department of Child and Adolescent Psychiatry, Bakirkoy Prof. Dr. Mazhar Osman Training and Research Hospital for Mental Health and Nervous Disorders, Istanbul, Turkey; ^6^Developmental Medicine Center, Boston Children’s Hospital, Harvard Medical School, Boston, MA, United States

**Keywords:** autism spectrum disorder, comorbidity, prevalence, epidemiology, child, adolescence, psychiatric

## Abstract

Psychiatric comorbidity in autism spectrum disorder (ASD) is a subject of critical scientific importance, affecting the quality of life, prognosis, and functional outcomes. The prevalence of psychiatric disorders vary considerably according to variables such as index subject characteristics, study setting, sampling frame, diagnostic methods used, as well as country of geographic origin. To date, most studies comprise clinical or treatment referral samples in tertiary care or subjects enrolled in clinical trials and genetic cohort collections. Such samples carry the potential for overestimation of both the frequency and severity of psychiatric comorbidity. A systematic literature search was performed using PubMed and Web of Science databases restricted to population-based study publications in the English between May 1, 2015, and May 31, 2020. A comprehensive keyword list was generated to investigate co-occurrence of psychiatric disorders in children and adolescents with ASD. A wide range of DSM-5 based disorders such as anxiety, mood, ADHD, intellectual disability/intellectual developmental disorder, eating/feeding, gender dysphoria and sleep-wake disorders were assessed. Initial search revealed a total of 1674 articles after removal of duplicates. Two independent researchers conducted a parallel-blinded screening process to identify the eligible studies based on titles and abstracts; 39 studies were analyzed in the current review. The main findings show prevalence estimates of 22.9% (95% CI: 17.7- 29.2) for intellectual disability; 26.2% (22-31) for attention-deficit hyperactivity disorder; 11.1% (8.6-14.1) for anxiety disorders; 19.7% (11.9-30.7) for sleep disorders; 7% (5.2- 9.3) for disruptive disorders; 2% (1.3- 3.1) for bipolar disorders; 2.7% (1.8- 4.2) for depression; 1.8% (0.4–8.7) for obsessive-compulsive disorder; and 0.6% (0.3–1.1) for psychosis. Psychiatric comorbidity in population-based studies is lower than in clinical and referred samples. However, our results also indicate that the frequency of psychiatric comorbidity in children and adolescents with ASD in the population context is considerable, without the influence of referral bias implicit in clinical and treatment samples. There is a need for better targeted diagnostic tools to detect psychiatric comorbidity in children and youth in future population-based studies, as an essential component in providing care as well as new insights into the nature and mechanisms of its underlying associations.

**Systematic Review Registration:**

[https://www.crd.york.ac.uk/prospero/], identifier [CRD42021234464].

## Introduction

Autism Spectrum Disorder (ASD) is a neurodevelopmental disorder characterized by persistent deficits in social communication and social interaction across multiple contexts and restricted, repetitive patterns of stereotyped behaviors, interests, or activities. The symptoms of ASD emerge in early childhood and usually remain lifelong (1). The prevalence of ASD has been increasing globally, ranging from 18.5/1000 among children aged 8-years in the United States to a median estimate of 6.2 (3-12.3)/1000 in Europe (2–5).

A prominent concern is the high frequency of cooccurring psychiatric disorders in children and adolescents with ASD that far exceed chance expectations. The data derived from clinical and treatment referral populations cannot be used to determine the population’s experience with psychiatric comorbidity which reflects the true relative risk of one or more disorders co-occurring in the presence of an index disorder in the population (6). There are varying forms in which comorbidity can be understood. It may represent an elevated frequency of co-occurring disorders at a point in time (*cross-sectional*), it may be measured over a specified duration (*period*), or across the lifespan of an individual (*lifetime*) (7). Three additional notions in which comorbidity can be understood include: (1) *pathogenic* in which an index condition leads to another considered to be etiologically related; (2) *diagnostic* based on shared symptoms that are not specific to a single disorder but overlap, leading to misclassification of symptoms. Such diagnostic challenges are particularly relevant in conditions such as distractibility in attention deficit hyperactivity disorder (ADHD) and ASD, and anxiety and sensory phenomena in ASD; and (3) *prognostic* comorbidity in which an index condition predisposes to, or is predictive of, development of other psychiatric disorders, e.g., the emergence of depressive disorders associated with environmental trauma-sensitive experiences.

It is now commonly accepted that characteristics of psychiatric comorbidity in ASD can change the affected person’s course and prognosis, quality of life, as well as functional outcomes across the lifespan. Given the highly heterogeneous nature of ASD, the comorbid psychiatric conditions may share overlapping risk factors and the pattern of comorbidity may itself constitute therapeutically meaningful subtypes. Knowledge gained in population based estimates of comorbidity is therefore not only practically important in tailoring interventions according to the distribution of psychiatric burden in ASD in the population, but understanding of psychiatric comorbidity is a topic of critical scientific importance.

Among psychiatric conditions commonly noted to have elevated frequencies in persons with ASD are anxiety disorders, ADHD, disruptive/impulse-control/conduct disorders, depres- sive as well as disruptive mood dysregulation disorders, as well as a multitude of challenging behaviors including stereotyped movements with self-injury, obsessions-compulsions, as well as sleep-related disorders (8–10). Historically, literature on ASD has shown a high rate of comorbidity with intellectual developmental disability (intellectual disability) (ID) (7, 11), but varying levels of intellectual impairment, as well as language development are now also envisioned as accompanying “specifiers” of ASD diagnosis (1).

In the current literature, the prevalence rates of psychiatric disorders vary considerably according to variables such as sample characteristics, study setting, diagnostic method, as well as country of origin, among others (7–9). The high heterogeneity in comorbid psychiatric conditions in ASD, as well as the heterogeneity of ASD itself makes the elucidation of the nature of psychiatric comorbidity in ASD a challenging task. It is therefore not surprising that a recent review of pooled data from both clinical and population-based studies by Lai et al. found that the prevalence of ADHD in ASD ranged between 4 - 63%, and that of anxiety disorders in ASD ranged between 2 - 48% (7–9). Such variations in co-occurrence of psychiatric disorders make it difficult to generalize and understand the topography of psychiatric comorbidity in persons with ASD. The situation is particularly salient in that the majority of studies on psychiatric comorbidity in ASD, to date, comprise clinical and referred samples often in tertiary care or rehabilitation centers, or among subjects with ASD enrolled in clinical trials and cross-sectional studies. Such ascertainment models carry a potential for overestimation of both the frequency and severity of psychiatric comorbidity with risk for spurious associations. An interesting pattern is that more recent studies have been reporting lower psychiatric comorbidity than older studies (7–9).

A better understanding of the relationship between diagnostic constructs, prognostic insight, and preventive treatment approaches can be gained by assessment of population-based psychiatric comorbidity in ASD. Therefore, the primary aim of this paper is to review the post DSM-5 era published population-based studies ‘in the English language’ utilizing valid structured measures of ASD in children and adolescents ages 6-18 years. A secondary aim is to investigate differences in rates of psychiatric comorbidity comparing child and adolescent age groups, 6-12, and 12-18 years, respectively.

## Materials and Methods

### Search Strategy and Methodological Quality

In the current review, The Preferred Reporting Items for Systematic Reviews and Meta-Analyses (PRISMA) guideline was followed (12). The review was registered on PROSPERO with registration number CRD42021234464. We have conducted an electronic search for published articles from 05/01/2015 to 05/31/2020 in PubMed and Web of Science databases. The search was limited to publications in the English language. A comprehensive keyword list was used to include original research articles investigating the co-occurrence of psychiatric disorders in children and adolescents with ASD. The keywords included terms from standard classifications such as DSM-5 as well as broader terms (Supplementary Material in Supplement A). The same strategy was used in both databases.

The authors TM and HAG conducted a parallel blinded screening process to identify the eligible studies based on titles and abstracts. The publications which were included by both authors were selected for full-text reviewing while the publications which were excluded by both researchers were removed and publications which were included by only one of the researchers were discussed on a case-by-case basis for the inclusion (13). Further manual screening of the reference lists of eligible studies and reviews was performed to find further studies related to the topic.

### Study Selection and Data Extraction

**FIGURE 1:**
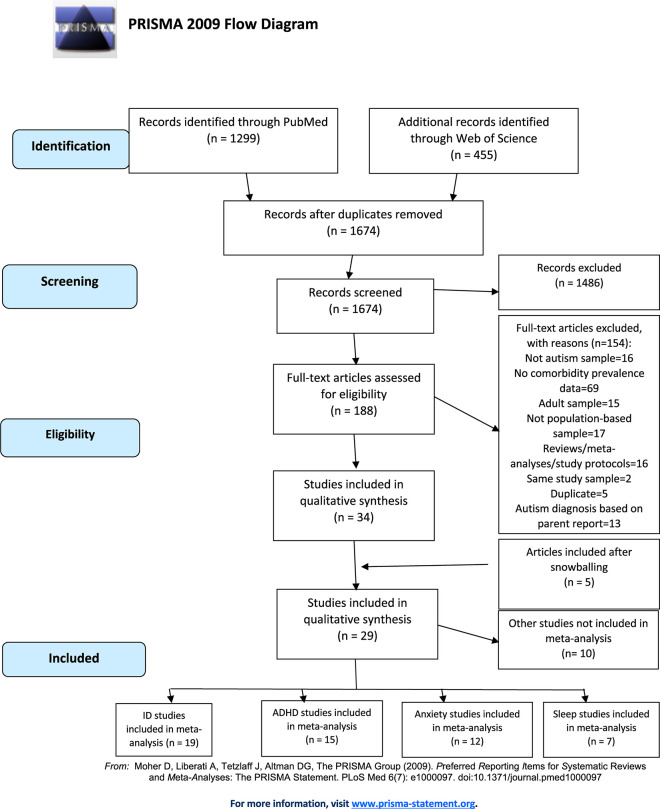
Flow diagram for identification of population-based studies on psychiatric comorbidities in autism spectrum disorders.

The eligibility criteria for the inclusion of an article were to have a population-based sample with a primary diagnosis of ASD; assessment of a wide range of DSM-5 based disorders such as anxiety, ADHD, ID, disruptive behavior disorder, bipolar disorder, depression, obsessive compulsive disorder, psychosis, eating/feeding disorder, gender dysphoria, and sleep-wake disorder. The age range of the studies was limited to index subjects with ASD who were 6-18 years old, however, articles with a broader age range were also included if the age group stratification and separate sample size and prevalence values were available (14, 15). Clinical trials, experimental studies, reviews, and studies primarily of well-recognized complex genetic/rare conditions, as well as neurological disorders such as epilepsy and metabolic disorders were excluded. The studies where the diagnostic methodology for the ASD or ID diagnosis was not explained or where the diagnosis of ASD was not made according to a structured DSM-IV/5 or ICD 9/10 criteria during the identified review period, or in which subjects were recruited according to parent-report with presumed diagnosis of ASD were excluded. The studies investigating health insurance databases or medical registries were only included if the relevant diagnostic criteria and pathways for diagnosis were explained in detail. The studies where the comorbidities were defined as categorical disorders (e.g., F20-29 or “emotional disorders”) rather than specific diagnoses were also excluded. The studies which represent only clinical samples, only adult samples were also excluded. A flow diagram of the search strategy and selection process is presented in Figure 1.

All included articles were read carefully and data related to the current review was identified and extracted by the authors TM, HAG, AOM, BE, and MC. Extracted data were re-checked by TM, HAG, and AOM. Case definitions included “any”, “all”, or “at least one” disorder for every diagnostic category using DSM-IV, DSM-5 or ICD-9/10; for example “any ADHD”, “all ADHD”, or “at least one ADHD”.

A standard form was used to extract data from studies including following variables: author, brief description of the study, study design, dataset, sampling method, country, total sample size, ASD sample size, age range, mean age when reported, male to female ratio, diagnostic tools for ASD, ID and the comorbidities, the prevalence of ASD, the prevalence of ID and relevant comorbidities, specifiers for the relevant comorbidity if reported. ID severity data were collected when reported and presented on the Supplement C in Supplementary Table, however, these data were not analyzed due to a small number of applicable studies.

Studies were coded as ‘child’ or ‘adolescent’ if the age range was limited to childhood or adolescence. This coding was not possible for some studies as they had a broad age range without stratification. The country data were categorized according to the continent they belong to. When the study reported the prevalence according to the age, country or sample source, the data were included into the analyses as separate study groups with sample size, age and prevalence data. Data from the studies using the same cohort sample or database (e.g., Swedish Twin Cohort, Autism Network Database) were carefully examined for overlapping samples or data.

### Statistical Analysis

All statistical analysis has been conducted using Comprehensive Meta-analysis software version 2, licensed to Koç University. Variance and heterogeneity in between the study subgroups included in each analysis have been analyzed using the Q value, p value and I^2^ statistics. Extracted data for sample size and events rate from each study and study subgroup have been used for meta-analysis statistics using a random effects model, due to heterogeneity. A forest plot has been generated for visualization of the major outcome variables: ID, ADHD and anxiety disorders. Publication bias has been investigated using the Egger’s test and Begg and Mazumdar rank correlation Kendall’s tau without continuity correction for analysis of ID, ADHD, and anxiety disorders studies at all pediatric populations (16, 17). For the other outcome variables, publication bias statistics have not been assessed due to low number of studies in each analysis.

## Results

### Description of Included Studies

The initial search revealed a total of 1674 articles after the removal of duplicates. EndNote reference manager ver. X7.4 (Philadelphia, PA, United States) software merged the search results and removed duplicate records. Following PRISMA guidelines the full texts of the potentially relevant reports were retrieved and examined for study compliance with the eligibility criteria. Following the abstract screening, a total of 188 full-texts were retrieved to be evaluated in the full-text review process. The articles with only adult samples, clinical samples, studies from single centers, review articles were excluded. The articles which don’t have ASD-only samples or don’t have population based data were also excluded. Studies which report comorbidity data without prevalence were excluded (e.g., reporting mean IQ score or mean scale scores of the total sample). In case that more than one article consists of the same sample, the article with the most recent data was included. When the ASD diagnosis is not made according to DSM or ICD criteria, or it is based on parent-reports or the diagnostic methodology is not clear, the article was excluded. In the full-text review step, 34 studies, and after screening the references of relevant articles, a further 5 studies were found eligible to be analyzed in the current review (Figure 1-flow chart). While the publication date of the studies dated back to 2015 due to search criteria, the studies had data since 2002.

Twenty nine studies consisting of ID, ADHD, anxiety disorders, disruptive behavior disorders, bipolar disorder, depressive disorders, OCD, psychotic disorders and sleep disorders prevalence were analyzed. Seven studies had subgroups which consisted of different countries, ages or registry samples (Figure 1) (5, 8–23). There were 33 study groups from 19 studies for ID (3, 5, 14, 18, 19, 21, 22, 24–34), 21 study groups from 15 studies for ADHD (14, 18, 19, 21, 27, 28, 31, 35–41), 19 study groups from 12 studies for anxiety (14, 18, 19, 21, 27, 28, 30, 36, 37, 39–41), 16 study groups from 10 studies for disruptive behavior disorders (14, 18, 19, 26, 27, 35–37, 39, 40), 12 study groups from 6 studies for bipolar disorders (14, 18, 19, 27, 30, 42), 12 study groups from 6 studies for depressive disorders (14, 18, 19, 27, 30, 40), 8 study groups from 5 studies for OCD (19, 27, 30, 35, 41), 7 study groups from 4 studies for psychotic disorders (18, 27, 30, 42) and 13 study groups from 8 studies for sleep disorders (18, 20, 23, 27, 36, 39, 40, 43) analyses (For the full list of studies: Supplementary Material in Supplement C). Ten studies consisting of other psychiatric comorbidities such as, suicide, bullying, trauma, tic disorder, gender identity disorder and enuresis/encopresis were not included in the meta-analysis due to the small number of studies, instead they were presented on Supplementary Material in Supplement C.

### Risk of Bias and Quality Assessment

Subgroups analyzed for ID comorbidity in all pediatric population groups did not reveal a publication bias as assessed by Egger’s test (Egger’s regression coefficient (CI): 1.2 (−11.6, 14), *p* value = 0.85) and Kendall’s tau (tau = 0.0011, *p* = 0.92). On the other hand, subgroups analyzed for ADHD comorbidity at all pediatric population groups revealed a publication bias when assessed by Egger’s test (Egger’s regression coefficient (CI): −22 (−32.45, −11.46), *p* value = 0.0003), however Kendall’s tau (tau = −0.19, *p* = 0.22) was not significant. Subgroups analyzed for anxiety disorders comorbidity at all pediatric population groups also did not reveal a publication bias as assessed by Egger’s test (Egger’s regression coefficient (CI): −11 (−21.5, −0.05), *p* value = 0.05) and Kendall’s tau (tau = −0.19, *p* = 0.25).

### Psychiatric Comorbidities in Children and Adolescents With Autism Spectrum Disorders

**FIGURE 2:**
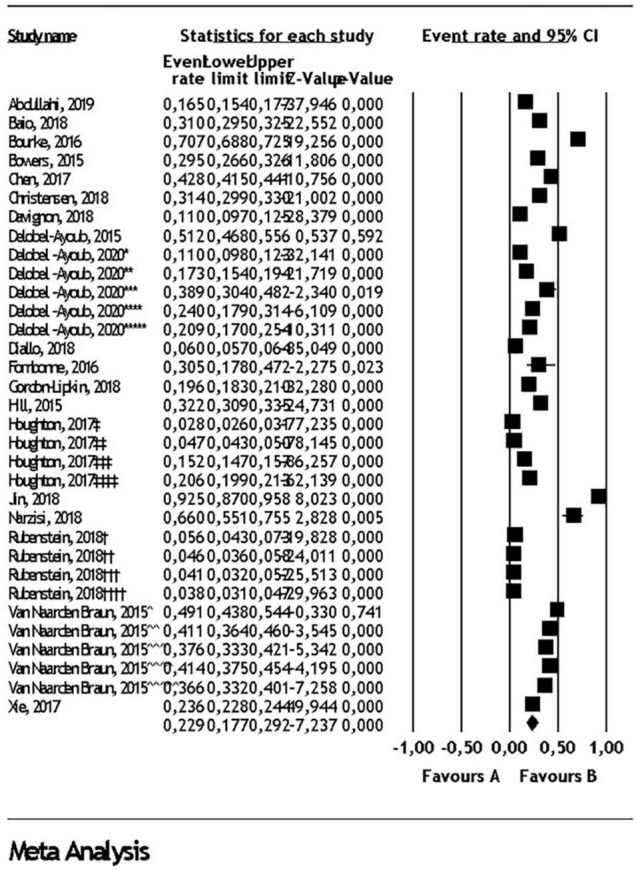
Intellectual disability comorbidity in autism spectrum disorder. Diamond represents the random effect model analysis result for the meta-analysis of 33 subgroups given in 17 studies (*: Denmark (2006-2008), **: Finland (2006-2008), ***: Southwest France (2007), ****: Southeast France (2007), *****: Iceland (2006-2008), †: 2002 ADDM data, ††: 2006 ADDM data, †††: 2008 ADDM data, ††††: 2010 ADDM data, ‡: 5-11 years old in commercial data, ‡‡: 12-17 years old in commercial data, ‡‡‡: 5-11 years old in Medicaid data, ‡‡‡‡: 12-17 years old in Medicaid data, ^: 2002 MADDSP data, ^^: 2004 MADDSP data, ^^^: 2006 MADDSP data, ^^^^: 2008 MADDSP data, ^^^^^: 2010 MADDSP data).

**FIGURE 3:**
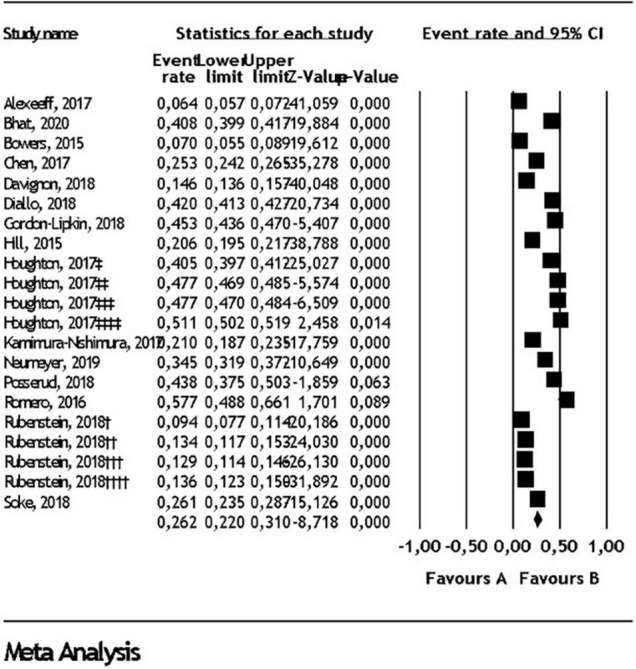
Attention deficit/hyperactivity disorder comorbidity in autism spectrum disorder. Diamond represents the random effect model analysis result for the meta-analysis of 21 subgroups given in 15 studies (†: 2002 ADDM data, ††: 2006 ADDM data, †††: 2008 ADDM data, ††††: 2010 ADDM data, ‡: 5-11 years old in commercial data, ‡‡: 12-17 years old in commercial data, ‡‡‡: 5-11 years old in Medicaid data, ‡‡‡‡: 12-17 years old in Medicaid data).

**FIGURE 4:**
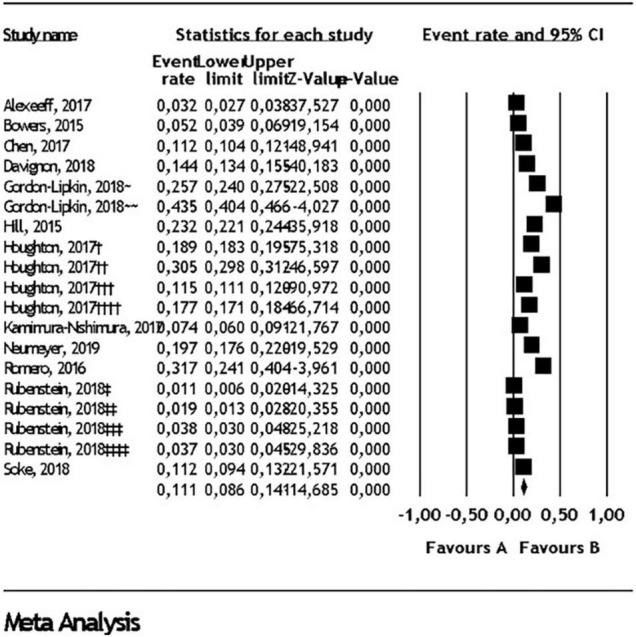
Anxiety disorder comorbidity in autism spectrum disorder. Diamond represents the random effect model analysis result for the meta-analysis of 19 subgroups given in 11 studies (†: 5-11 years old in commercial data, ††: 12-17 years old in commercial data, †††: 5-11 years old in Medicaid data, ††††: 12-17 years old in Medicaid data, ∼ 6-11 years old, ∼∼ 12-17 years old, ‡: 2002 ADDM data, ‡‡: 2006 ADDM data, ‡‡‡: 2008 ADDM data, ‡‡‡‡: 2010 ADDM data).

Random effect analysis for each comorbidity in all pediatric populations, children and adolescents, in addition to heterogeneity for each analysis can be found in Table 1. Prevalence of ID in all pediatric populations was found as 22.9% (95% CI: 17.7- 29.2) (Figure 2). Prevalence of ADHD in all pediatric populations was found as 26.2% (Figure 3) and prevalence of anxiety disorders in all pediatric populations was found as 11.1% (Figure 4). All statistical analysis groups showed high heterogeneity as shown by I^2^ value higher than 84.

**TABLE 1:** Population-based psychiatric comorbidity studies in children and adolescents with autism spectrum disorders.

					Heterogeneity analysis	
					
Comorbidity	Study Population	Number of study groups	Total number of patients	Prevalence (95% CI)	Heterogeneity I2	Cochran Q, p value
Intellectual disability	All pediatric population	33	136155	22.9 (17.7- 29.2)	99.76	13430, < 0.001
	Children	22	58849	21.4 (15.2- 29.2)	99.57	4886, < 0.001
	Adolescent	4	35540	15.6 (5.8- 35.8)	99.91	3502, < 0.001
ADHD	All pediatric population	21	126951	26.2 (22-31)	99.72	7164, < 0.001
	Children	10	49617	18.4 (12.3- 26.6)	99.76	3716, < 0.001
	Adolescent	3	32163	35.4 (21.9-51.7)	99.86	1477, < 0.001
Anxiety disorders	All pediatric population	19	97791	11.1 (8.6-14.1)	99.56	4117, < 0.001
	Children	11	51688	7.8 (5.3- 11.3)	99.4	1667, < 0.001
	Adolescent	5	38746	21.5 (14-31.6)	99.73	1468, < 0.001
Sleep Disorders	All pediatric population	13	133233	19.7 (11.9-30.7)	99.91	13381, < 0.001
	Children	9	99849	26.5 (15.4 -41.6)	99.91	8942, < 0.001
	Adolescent	3	32163	6.6 (4.5-9.5)	98.67	151, < 0.001
Disruptive Behaviors	All pediatric population	16	88855	7 (5.2- 9.3)	99.48	2900, < 0.001
	Children	10	48770	6.1 (3.9-9.2)	99.36	1421, < 0.001
	Adolescent	4	37781	9.8 (5.3- 17.5)	99.77	1292, < 0.001
Bipolar Disorder	All pediatric population	12	91052	2 (1.3- 3.1)	99.39	1796, < 0.001
	Children	7	44209	1 (0.5- 1.8)	98	300, < 0.001
	Adolescent	5	46843	4.8 (2.9- 7.7)	99.37	642.5, < 0.001
Depression	All pediatric population	12	85901	2.7 (1.8- 4.2)	99.53	2331, < 0.001
	Children	8	48120	1 (0.5- 1.7)	98.11	371.16, < 0.001
	Adolescent	4	37781	12.7 (11-14.7)	96.46	84.85, < 0.001
Obsessive Compulsive Disorder	All pediatric population	8	11914	1.8 (0.4- 8.7)	99.16	837.5, < 0.001
	Children	7	7791	1.7 (0.2- 11.2)	98.98	587.5, < 0.001
	Adolescent	-	-			
Psychosis	All pediatric population	7	79055	0.6 (0.3- 1.1)	97.84	277.8, < 0.001
	Children	3	37830	0.2 (0.1-0.4)	84.75	13, 0.001
	Adolescent	4	41225	1.1 (0.6- 1.8)	96.31	81.29, < 0.001

### Other Studies Not Presented in the Tables

Reviewed studies included also prevalence rates for some additional comorbidity such as suicide, bullying, trauma, tic disorder, gender identity disorder and enuresis/encopresis (14, 15, 30, 35, 44–51). These comorbidities were not included in the meta-analysis due to the low number of studies, however they are presented with details in the Supplement C in Supplementary Table for interested readers.

## Discussion

In this meta-analysis, we have reported the rates of psychiatric comorbidity in ASD, specifically for population-based research studies. Overall, the prevalence figures found in our meta-analyses were lower than other published studies in the literature (8). This finding can be explained in terms of number of approaches to the present analyses. First, we examined population-based studies only, so data from clinic-based studies conducted with a high number of complex patients were not represented in our analyses. Second, we analyzed studies using the children and adolescents ages 6-18, and comorbidities emerging in adulthood were not represented. Third, we included studies investigating population-based ASD prevalence. Finally, we included articles from the last five years, published in the post-DSM-5 period, to allow more uniform research methodology and greater experience garnered by investigators in recent years.

As in other ASD comorbidity studies, the rates of heterogeneity in our analysis results were quite high. The use of different diagnostic tools for both ASD and comorbid conditions seems to be one of the important factors explaining the heterogeneity. The studies we analyzed had various diagnostic tools, which may have led to measurement error. This assumption reflects the need for diagnostic tools specific to ASD. The Autism Comorbidity Interview (ACI) is a semi-structured interview that utilizes the Kiddie Schedule for Affective Disorders and Schizophrenia (K-SADS) with adaptation to increase validity in the ASD population (52). Other factors contributing to the heterogeneity could be age, varying sample sources across different countries, and inclusion of insurance and research registries.

### Comparison of Comorbidity Pattern in Children and Adolescents

Our review shows a critical gap in the field for population-based comorbidity studies focusing in particular on adolescents with ASD. Most of the studies include school-aged children and do not mirror the needs of older patients. Our meta-analysis included 5 studies involving adolescents, of which 3 were derived from large integrated health care delivery systems and insurance cohorts (14, 18, 27). Although these databases contain extensive registers, they are also based on clinical information derived from medical records. Nonetheless these were total population samples of insurance membership even though they reflect self-contained samples. Nevertheless, these data sources need to be more carefully interpreted and carefully designed future population-based studies are needed in order to make better generalizations about adolescent psychiatric comorbidity in ASD.

Nonetheless, our findings demonstrate that adolescents with ASD have higher comorbidity for psychopathology, including ADHD, anxiety, disruptive behavior, bipolar, depression, OCD and psychosis compared to children with ASD, in line with the most current meta-analysis on ASD comorbidity (9). Higher prevalence in adolescents might be due to the late-onset and older age diagnosis of most psychopathologies. Additionally, adolescence is a time period when individuals struggle with multiple physical, emotional and social changes. This might make adolescents more vulnerable to these mental health problems even in the general population. Finally, there is a temporal difference among adolescents and children that may reflect qualitative differences in the year of diagnosis of ASD across age groups. The support for this is reflected in the ID comorbidity data among children compared to adolescents (23.2 vs. 15.6%, respectively), suggesting that the adolescents comprise a higher functioning subgroup where psychiatric comorbidity can be more readily identified.

An exception from the current literature on ASD comorbidity involved the prevalence findings regarding ADHD. Our results from 2 studies (18, 27) on the prevalence of ADHD found similar figures between adolescents and children, 21.9 vs. 18.4%, respectively. This again may reflect the longer lead period with greater likelihood of identification of ADHD among adolescents compared to younger children with ADHD being highly prevalent among both age groups. Finally, sleep comorbidities were higher among children (26.6%) compared to adolescents (6.6%), consistent with prior observations that sleep problems and sleep-related complaints are usually higher in earlier developmental stages (53).

### ID Comorbidity

The prevalence of ID comorbidity in ASD was 22.9% according to our analysis of 33 study groups (3, 5, 14, 18, 19, 21, 24–34, 54). In the ID analyses, studies used varying diagnostic measures which could not be used for the survival analysis to assess the effect of diagnostic tools on prevalence. The studies were therefore pooled to conduct meta-analyses using random effect models. The measures of cognitive assessment were highly variable from study to study, which resulted in remarkable heterogeneity. For instance, Narzisi and Jin reported WISC-4 as the measurement tool, which resulted in relatively high rate of ID prevalence (25, 32). Most other studies reported that they classified patients as ID based on fullscale IQ scores below 70 without mention of specific test measures. Furthermore, the diagnosis of ID did not conform to the current DSM-5 definition, as IQ scores do not incorporate overall adaptive functioning and such analyses cannot point to the severity level of ID based on IQ cut off scores alone. Additional considerations have included, child’s ability to cooperate with the IQ testing that is known to be characteristically difficult due to poor testability of subjects with ASD, with significant variance of cognitive testing results (55). Further studies are needed incorporating measures of adaptive functioning in ASD patients with or without ID, especially those with unusual cognitive profiles and patterns of learning (55). The adaptive functioning was not reported in almost any of the studies included in the current meta-analysis and ID severity was not examined.

### Attention Deficit Hyperactivity Disorder Comorbidity

The prevalence of ADHD in ASD varied from 28 to 83% across studies. Studies using DSM-IV reported lower rates of ADHD in contrast to ones using DSM-5 criteria. On average a quarter of subjects with ASD had ADHD comorbidity based on current population-based studies we included (14, 18, 19, 21, 27, 30, 31, 35–41). The pattern of behavior in children and adolescents including inattention and or hyperactivity-impulsivity may overlap between the two conditions reflecting diagnostic comorbidity, as well as impairments in activities of daily living, as well as social adaptation and behavior problems in ADHD that has prognostic significance for learning in children and adolescents with ASD. The prevalence of comorbid ADHD was reported in three reviews in which the values were comparable to the current study (9, 56, 57). Lugo-Marín and colleagues found the pooled prevalence of ADHD at 25.7% among 24, 511 individuals (57). In another review by Lai and colleagues, the pooled prevalence among 210, 249 participants with ASD was 28%, close ratio to the current analysis (9).

### Depression and Anxiety Disorders Comorbidity

The study findings support the view that social communication problems, sensory aversions, disruptive emotional dysregulation, inflexible adherence to routines and difficulty in tolerating change may predispose children and adolescents with ASD to higher frequency of internalizing disorders (58). We included 12 study groups (14, 18, 19, 21, 27, 30, 36, 37, 39–41) and found depression comorbidity to be 2.7%, which is lower than previously reported. The prevalence of anxiety comorbidity in ASD was 11.1% among the 19 study groups. Anxiety subcategories such as general anxiety disorder, and social phobia, were not reported in most studies, and could not be included, and was again lower than the comorbidity results reported by other meta-analyses (9, 59). Notably, the underestimation of comorbidity findings for depression and anxiety may be related to three salient factors. First, evaluating internalizing disorders in children and adolescents with ASD is challenging and often unrecognized due to the difficulties in expression of their feelings and thoughts. Second, the internalizing conditions are in themselves more challenging to elicit in larger scale population-based studies where comorbid emotional conditions may not be examined as readily as in diagnosing ADHD. Finally, the use of reliable anxiety scales had limited use in the studies included, with likelihood of underreported or missed diagnosis. A small number of measurement tools have been developed and can lead to better characterization of comorbidity related to anxiety and depression in ASD (60, 61).

### Disruptive Behavior Disorders Comorbidity

According to the results of three reviews in the literature, the prevalence of disruptive behavioral disorders in ASD ranged from 12 to 48% (9, 62, 63). Although we included 19 study groups (14, 18, 19, 26, 27, 35–37, 39, 40) with a wide range of definitions of disruptive behaviors in our analysis such as oppositional defiant disorder, conduct disorder, and disruptive behavior problems (see Supplementary Material) the comorbidity finding of 7% is again lower than previously reported. This again may reflect population-based feature of included study groups. The studies included also reflect lower comorbidity with ID which in turn may reflect a lesser degree of challenging disruptive behaviors, in particular among adolescents. Furthermore, these conditions attenuate with advancing age.

### Psychosis and Bipolar Disorder Comorbidity

The observed comorbidity with psychosis in ASD was overall quite rare (0.6%), with doubling of the level (1.1%) among adolescents (14, 18, 19, 27, 30, 42). The onset of psychotic symptoms is likely to emerge in late adolescence and adulthood than in early childhood (64). Nonetheless, the comorbidity among adolescence in population-based study is not low. Behavioral phenotypes of known genetic conditions, e.g., 22q11 deletion syndrome, may lead to greater likelihood for identification of psychosis.

Two prior reviews evaluated the comorbidity of bipolar disorder with ASD. First study reported prevalence between 6% to 21.4% (65); second study reported a prevalence of 5% (95% CI: 3-6) (9). We involved 12 study groups and found 2% prevalence among 91,052 child and adolescent populations and the lower level may reflect the younger age dominance of our sample.

### OCD Comorbidity

We found a prevalence of 1.8% in our meta-analysis lower than prior reports in the literature (19, 27, 30, 35, 41). First, this may reflect greater awareness of diagnostic misclassification of restrictive, repetitive behaviors, interests, and activities, as connoting OCD symptoms. Furthermore, the restrictive, repetitive behaviors in children and adolescents with ASD tend to be more likely to be ego-syntonic compared to the ego-dystonic nature of OCD symptoms, although admittedly this is not a reliable distinguishing feature. Second, the younger age dominance is less likely to lead to emergence of prototypical OCD symptoms making diagnosis less likely. On the other hand, Leyfer and colleagues, a decade earlier, found that 37% of their sample met criteria of OCD (52). In a different study by Simonoff and colleagues, the assessment tool did not allow for caregivers to make this inference and the prevalence of OCD was estimated as 8.2% (66). Studies using DSM-IV criteria were also included in our meta-analyses; in these studies, OCD diagnosis may have been represented as anxiety disorders, which may lead to a lower prevalence of OCD.

### Sleep Disorders Comorbidity

While parents of children with ASD report sleep problems from 50 to 80%, this rate has buried between 9% and 50% in normally developing children (67). Sleep problems contribute to heightened daytime cognitive, adaptive and behavioral problems among children and adolescents (68). The prevalence of sleep disorder related comorbidity in ASD was 19.7% in the current study based on analysis of 13 sleep study groups (18, 20, 23, 27, 36, 39, 40, 43). Subtypes of sleep problems were not reported in most studies and were not included. Population-based studies reported mostly parent-reported sleep disorders and did not involve subjective assessments of sleep. A systematic review and meta-analysis of sleep-related problems in ASD including both subjective and objective sleep measures in children, affirmed that children with ASD presented with significantly higher level of sleep impairments, quantified both by subjective and objective parameters compared to typically developing children (69).

### Strengths

The current meta-analysis represents a comprehensive review of the population-based prevalence of most often reported psychiatric comorbidities in children and adolescents with ASD. Nine psychiatric comorbidities were selected. Only population-based studies and large-scale insurance registries were included. Clinic or school samples, or samples where diagnosis was not confirmed by measurement tools, or reflected only informant reports, were excluded. The study was restricted to studies published in the post-DSM-5 period with possibly greater diagnostic homogeneity, and relevance to the current period. Studies included different geographic regions, albeit mostly from high income given the predominance of available data. Nonetheless, the results provide an inclusive global perspective of psychiatric comorbidity globally. Although, our estimates were lower than in clinical samples, the frequency of psychiatric comorbidity in the present analysis are devoid of referral bias.

### Limitations

The findings of the present study should be considered in the context of number of limitations. First, the studies predominantly reflect the situation in high-income countries and hinders the generalizability of the results Second, most of the studies didn’t have a control group of typically developing children, so an accurate comparison to population prevalence estimates of psychiatric disorders can not be made. Third, the studies garnered did not report language skills, verbal ability, or communication skills, which may lead to underestimation of psychiatric comorbidity. We couldn’t analyze their contribution to prevalence. Although, we restricted selection to a publication date between 2015 and 2020, some of the samples in the studies ranged from 2002, resulting in presentation of less recent data and possible period and age effects that cannot be mitigated. Diagnostic criteria used in the studies included ICD-9, ICD-10, DSM-IV and DSM 5, and the measurement tools varied across the studies. The quality of the studies included in the meta-analysis was highly variable. Those limitations may have led to enhance the variability of the prevalence results. We analyzed adolescent comorbidity prevalence with data from very few studies, and although consistent with prior reports, our findings for adolescents nevertheless ought to be interpreted with caution.

## Conclusion

Our results indicate that the frequency of psychiatric comorbidity in children and adolescents with ASD in the population context is considerable, without the influence of referral bias implicit in clinical and treatment samples. There is paucity of studies examining psychiatric comorbidity in children in general, and in adolescents in particular. In future population-based studies, there is a need for better targeted diagnostic tools to detect psychiatric comorbidity in children, youth, as well as adults with ASD. This represents a major gap comparted to the time and careful attention given to diagnostic accuracy of ASD itself. Indeed, the disruptive, and preventable, burden of psychiatric comorbidity in children and adolescents with ASD is likely to be more restrictive in its educational and social participation impact, and more strongly influential on the level of well-being and quality of life of parents and families, than the severity level of the ASD diagnosis (6- Munir). The approach to understand psychiatric comorbidity in ASD also represents an essential component for providing insights into nature and mechanisms of its underlying associations and outcomes.

## Data Availability Statement

The raw data supporting the conclusions of this article will be made available by the authors, without undue reservation.

## Author Contributions

TM, HA, and KM were responsible for study design and contributed to data interpretation and article writing. TM, HA, BE, AÖ, and MC performed literature review and article search and data extraction. BE, MC, and AÖ contributed in article writing. HY performed statistical analyses, interpretation of the results and conceptualizing according to the current literature. TM, HA, HY, and KM was mainly responsible for study conceptualization and article writing. All authors personally revised and approved the final version of the manuscript.

## Conflict of Interest

The authors declare that the research was conducted in the absence of any commercial or financial relationships that could be construed as a potential conflict of interest.

## Publisher’s Note

All claims expressed in this article are solely those of the authors and do not necessarily represent those of their affiliated organizations, or those of the publisher, the editors and the reviewers. Any product that may be evaluated in this article, or claim that may be made by its manufacturer, is not guaranteed or endorsed by the publisher.
