# Supplementary material for: Population-Based Psychiatric Comorbidity in Children and Adolescents With Autism Spectrum Disorder: A Meta-Analysis
Source: Front Psychiatry. 2022 May 23;13:856208. doi: 10.3389/fpsyt.2022.856208 (PMC9186340; doi:10.3389/fpsyt.2022.856208)
Supplement: Supplementary file 1 [file Data_Sheet_1.docx]

**Supplement A:**

The search terms used were:

(((("autism spectrum disorder" OR "autistic traits" OR autism OR "autistic disorder") AND (child OR pediatric OR adolescen*)) AND (epidemiolog* OR "population study")) AND (ADHD OR ''attention deficit hyperactivity disorder'' OR ''anxiety disorder'' OR depressi* OR schizo* OR ''bipolar and related disorder'' OR bipolar OR ''obsessive-compulsive and related disorder'' OR OCD OR disruptive OR impulse-control OR conduct OR sleep–wake OR ''sleep disorder'' OR ''trauma and stressor-related disorder'' OR PTSD OR ''substance-related and addictive disorder'' OR ''substance use'' OR ''gender dysphoria'' OR ''disruptive mood dysregulation syndrome'' OR DMDD OR food OR eating OR ''social phobia'' OR ''social anxiety'' OR ''oppositional defiant disorder'' OR ODD OR ''intellectual disability'' OR ''mood disorder'' OR ''feeding and eating disorder'' OR ''feeding disorder'' OR ''eating disorder'' OR ''trauma and stress related disorder'' OR ''communication disorders'' OR ''tic disorders'' OR tourette OR ''elimination disorders'' OR ''nonorganic enuresis'' OR ''non organic enuresis'' OR ''non organic encopresis'' OR ''nonorganic encopresis'' OR enuresis OR encopresis OR ''manic disorder'' OR ''Panic disorder'' OR ''panic attack'' OR ''acute stress reaction'' OR ''conversion disorder'' OR ''somatoform disorder'' OR ''somatization disorder'' OR insomni* OR hypersomni* OR sleepwalk OR ''sleep walk'' OR somnambulism OR ''sleep terror'' OR ''night terror'' OR nightmare OR ''impuls* disorder'' OR trichotilloman* OR ''gender ident* disorder'' OR ''speech disorder'' OR ''language disorder'' OR ''reading disorder'' OR ''spelling disorder'' OR ''arithmetic disorder'' OR scholastic OR ''hyperkinetic disorder'' OR mutism OR pica OR stutter* OR ''global developmental delay'' OR cataton* OR ''body dysmorphi* Disorder'' OR ''hoarding disorder'' OR ''hair pulling disorder'' OR excoriation OR ''skin picking disorder'' OR ''functional neurological symptom disorder'' OR ''rumination disorder'' OR ''food intak* disorder'' OR ''hypersomnolence disorder'' OR narcolep* OR parasomn* OR ''Restless Legs Syndrome'' OR ''intermittent* explosive disorder'' OR suicid* OR psychosis OR psychotic OR agoraphobia OR phobia OR ''anorex* nervos* '' OR bulimia OR bulimic OR ''binge eating disorder'')) AND (("2015/05/01"[Date - Publication] : "2020/05/31"[Date - Publication]))

Filters applied: Humans, English, Adolescent: 13-18 years, Child: 6-12 years.
