# Supplementary material for: Population-Based Psychiatric Comorbidity in Children and Adolescents With Autism Spectrum Disorder: A Meta-Analysis
Source: Front Psychiatry. 2022 May 23;13:856208. doi: 10.3389/fpsyt.2022.856208 (PMC9186340; doi:10.3389/fpsyt.2022.856208)
Supplement: Supplementary file 2 [file Data_Sheet_2.docx]

**Supplement B**

**List of Abbreviations and Acronyms**

**Acronyms:**

ACE – Adverse childhood experiences

IQ – Intelligence quotient

OME – Office of the Medical Examiner

PTE – Potentially traumatic events

TO –Typically developing

YO – years old

**Diagnosis:**

ADHD – Attention Deficit and Hyperactivity Disorders

ASD – Autism Spectrum Disorders

BID – Borderline Intellectual Disability

CD – Conduct Disorder

DD – Developmental Disorders

ID – Intellectual Disability

NDD – Neurodevelopmental Disorders

ODD – Oppositional and Defiant Disorder

PDD – Pervasive Developmental Disorder

**Datasets:**

ADDM – Autism and Developmental Disabilities Monitoring

AGRE – Autism Genetics Research Exchange

ALSPAC – Avon Longitudinal Study of Parents and Children

ASDEU – Autism Spectrum Disorders in the European Union

ATN – Autism Treatment Network

BCS – Bergen Child Study

CATSS – Child and Adolescent Twin Study in Sweden

IAN – The Interactive Autism Network

IBIS‐PH – Utah's Indicator‐Based Information System for Public Health

IDEA – Intellectual Disability Exploring Answers

KPNC – Kaiser Permanente Northern California

LKH – Dr. Leo Kannerhuis clinic

MADDSP – The Metropolitan Atlanta Developmental Disabilities Surveillance Program

MHS – Military Health System

NAMCS – National Ambulatory Medical Care Survey

NHAMCS – National Hospital Ambulatory Medical Care Survey

QICDSS – The Quebec Integrated Chronic Disease Surveillance System

SPARK – Simons Foundation Powering Autism Research

SSC – Simons Simplex Collection

STRIDE – Stanford Translational Research Integrated Database Environment

UPDB – Utah Population Database

URADD – Utah Registry of Autism and Developmental Disorders

Cros Sec – Cross Sectional studies

Pop Reg – Population-based/Registry-based studies

Prosp – Prospective studies

Retro – Retrospective studies

Sch Pop – School based population studies

**Assessment Tools:**

ADI-R – Autism Diagnostic Interview – Revised

ADOS – Autism Diagnostic Observation Schedule

ASSQ – Autism Spectrum Screening Questionnaire

BASC-2 PRS– Behavior Assessment System for Children, Second Edition, Parent Rating Scales

CAQ – Autism Spectrum Disorder Questionnaire

CBCL — Child Behavior Checklist

CSHQ — Children’s Sleep Habits Questionnaire

DCDQ – Developmental Coordination Disorder Questionnaire

DSM-4 - TR – Diagnostic and Statistical Manual of Mental Disorders, Fourth Revision, Text Revision

DSM-5 – Diagnostic and Statistical Manual of Mental Disorders, Fifth Revision

FFQ – Food frequency questionnaire

ICD-9-CM – International Classification of Diseases, Ninth Revision, Clinical Modification

ICD-10 – International Classification of Diseases, Tenth Revision

SCQ – Social Communication Questionnaire

SDQ – Strength and Difficulties Questionnaire

SRS – Social Responsiveness Scale

VABS – Vineland Adaptive Behavior Scales

WISC-R – Wechsler Intelligence Scale for Children - Revised Edition

WISC-4 – Wechsler Intelligence Scale for Children - Fourth Edition

WPPSI – Wechsler Preschool and Primary Scale of Intelligence

YSR – Youth Self-Report
