# Supplementary material for: Population-Based Psychiatric Comorbidity in Children and Adolescents With Autism Spectrum Disorder: A Meta-Analysis
Source: Front Psychiatry. 2022 May 23;13:856208. doi: 10.3389/fpsyt.2022.856208 (PMC9186340; doi:10.3389/fpsyt.2022.856208)
Supplement: Supplementary file 3 [file Data_Sheet_3.docx]

Supplement C: Supplementary tables

- All Abbreviations and Acronyms listed in the Supplement B

| **Table 1. Population-Based Study Characteristics of Intellectual Disability Comorbidity in Children with Autism Spectrum Disorder** | | | | | | | | | | | | | | |
| --- | --- | --- | --- | --- | --- | --- | --- | --- | --- | --- | --- | --- | --- | --- |
| **Author (year)** | **Aim of the study** | **Study design** | **Dataset** | **Inclusion Criteria** | **Country** | **Total (N)** | **ASD Sample** | | | **Diagnostic tools** | | **Prevalence of ASD** | **Prevalence of comorbid ID in ASD** | **Specifiers of ID** |
|  |  |  |  |  |  |  | n (ASD) | Age | M/F | ASD | ID |  |  |  |
| Delobel-Ayoub, et al. (2020) | To determine ASD prevalence in four European countries | Pop Reg | Nationwide health registry  ASDEU | Children born between 2006-2008 with a diagnosis of ASD before 31 December 2015 | Denmark  Finland  France-SW  France-SE  Iceland | 64.550  58.727  15.836  32.342  4500 | 2414  1347  113  154  363 | 7-9 | 3.9  3.3  5.4  4.0  4.4 | ICD-10 Codes for ASD | ICD-10 | 1.26 %  0.86 %  0.73 %  0.48 %  3.13 % | **11%**  **17,3%**  **38,9%**  **24%**  **20,9%** | 11.5 % male  19.4 % male  35.8 % male  20.3 % male  - |
| Narzisi, et al. (2018) | To estimate ASD prevalence in Italy | Sch Pop | Nationwide health registry ASDUE | Children, born between 2007-2009, living within the metropolitan area of Italy | Italy | 10.138 | 81 | 7-9 | 84%  5.2 | SCQ  ADI-R  ADOS | WISC-4 | 0.8 % | **41% Mild**  **25% Severe**  **34% Average** | - |
| Xie, et al. (2017) | To evaluate the risk of having ASD / ID with gestational age at birth | Longitudinal Register Linkage Cohort Study | Stockholm Youth Cohort | Children born between 1984 -2007 residing in Stockholm Country, Sweden | Sweden | 480.728 | 10.025 | 0-17 | 70.9  % | ICD-9 299,  ICD-10 F84  DSM-4 299 | ICD-9 317-319  ICD-10 F70-79  DSM-4 317-319 | 2.1 % | **23.6%** | Gestational age at birth, gender |
| Jin, et al. (2018) | To estimate the prevalence of ASD in Shanghai | Sch Pop | Shanghai multi-stratified cluster children sample | Children aged 3-12 years attending schools in the selected districts | China | 74.252 | 147 | 3-12 | 53.3 % | SCQ  DSM-5 | GESSEL (3-4 yo)  WPPSI  (4-6 yo)  WISC-R  (6-12 yo) | 0.83 % | **81.6%**  IQ below 40  **10.9 %**  IQ 40-70  **7.5 %**  IQ equal or greater than 70 | - |
| Fombonne, et al. (2016) | To estimate ASD prevalence in Mexico | Sch Pop | The Leon Survey | Children were born between Jan 1st and Dec 31th 2003 residing in Leon | Mexico | 12.116 | 36 | 8 | 80.6 %  4.1/1 | SRS  ADI-R  ADOS | Based on a clinical estimate derived from a review of cognitive data available | 0.87 % | **36.1 %**  Borderline IQ  **11.1 %**  Mild ID  **19.4%**  Moderate / Severe ID | Gender:  27.6% boys  42.9% girls |
| Davignon, et al. (2018) | To determine frequency of  medical and psychiatric conditions in a large population of  individuals with ASD | Electronic medical records cohort | KPNC data from 2013-2015 | 14-25 year old individuals as of January 1, 2014, members of KPNC for at least 9 months in each calendar year from 2013 -2015 | USA | 385051 | 1882 | 14-17 | 4:1 | ICD -9  ADOS | ICD - 9 | NA | **11%** for 14-17 |  |
| Christensen, et al. (2018) | To estimate the prevalence and characteristics of ASD among children aged 8 years | Pop Reg | ADDM | Children born in 2004, parents live in site-specific ADDM surveillance areas during 2012 | USA | 346.978 | 5063  (3353 had ID data) | 8 | 4.5:1 | **1th phase:**  ICD Codes  **2th phase:**  Clinician review based on DSM-4 TR and DSM-5 | Standardized tests IQ score < 70 = ID diagnosis | - | **31.4 %** ID  **24.5 %** BID  43.9 % Average or above the average | ID did not vary by sex, race/etnicity, with the exception of Georgia (ID in boys 87%, in girls 79%) |
| Rubenstein, et al. (2018) | To estimate trends in documented co-occuring conditions in ASD children | Pop Reg | ADDM | Children born in 1994, 1998, 2000, 2002, parents live in site specific areas in 2002, 2006, 2008, 2010 surveillance years | USA | 6379 | **2002**: 932  **2006**:1429  **2008**: 1666  **2010**: 2352 | 8 | 80.8 % | **1th phase:**  ICD Codes  **2th phase:**  Clinician review based on DSM-4 TR and DSM-5 | Standardized tests IQ score < 70 = ID diagnosis | **-** | 2002: **5.6 %**  2006: **4.6 %**  2008: **4.1 %**  2010: **3.8 %** | ID trends in different years |
| Baio, et al. (2018) | To estimate the prevalence of ASD in 2014 in USA | Pop Reg | ADDM | Children born in 2006 parents living in 9 specific areas for 2014 surveillance | USA | 325.483 | 2014: 5473  (only 3714 have ID data) | 8 | 3.2 (Arizona) to 4.9 (Georgia) | ICD  DSM-4  DSM-5 | Standardized tests IQ score < 70 = ID diagnosis | 1.68 | **31%** ID  **25%** BID  **44%** Average | ID did not differ by sex or race in any sites, differed by sex, boys are more likely than girls to have IQ more than 85. |
| Hill, et al. (2015) | To determine prevalence of unhealthy weight in ASD | Pop Reg  Cros Sec | Autism Speaks ATN from 2008-2013 | Children ages 2 to 17 with confirmed diagnosis according to DSM 4 and ADOS | USA and Canada | N= 13897  ASD n=5053 control group n=8844 | 5053 | 2-17 | M:4270  F: 783 | DSM-IV, ADOS, VABS 2 | clinical staff measured-IQ<70 | NA | **32.2%** |  |
| Abdullahi, et al. (2019) | To compare the ASD clinical profiles from migrant and non-migrant backgrounds | Pop Reg  Prosp | Western  Australian (WA) Register for Autism Spectrum Disorders | Children registered  between 1999 and 2017 who were younger than 18 years  of age at time of their diagnosis | Australia | 4776 | 3975 | 0-18 | 81.2 | DSM-4  DSM-5 | Bayley, Weschler Preschool and Primary Scale of Intelligence, WISC, Griffith Mental Development Scale, and Stanford Binet Intelligence Scale | NA | **16.5 %** |  |
| Houghton, et al. (2017) | To investigate the prevalence and length of prescriptions by age, psychiatric comorbidity for ASD | Insurance Data Cohort Study | Truven Health MarketScanVR Commercial Database and the Truven Health MarketScan Multi-State Medicaid Database | At least two claims for ASD with ICD-9 codes, of which at least one had to be recorded in 2014, >3 years old and non-Rett | USA | 46,943 commercially-insured and 46,696 Medicaid-insured subjects | Commercially insured**5–11:** 17,420 **12–17:** 15,086  Medicaid **5–11:**19,527  **12–17:** 12,954 | 5-11 12-17 | 80.47 % M between the ages 5-11  79.48 % M between the ages 12-17 | ICD-9 | ICD-9 | **Commercially Insured Data**: 30.588 (ASD), 13.952 (PDD), 2,403 (unspecified PDD)  **Medicaid Data:** 35,554 (ASD), 9,567 (PDD), 1575 (unspecified PDD) | **Commercially Insured Data**  **Ages 5-11:** 2.80  **Ages 12-17:** 4.66  **Medicaid Data**  **Ages 5-11:** 15.19  **Ages 12-17:** 20.58 |  |
| Delobel-Ayoub, et al. (2015) | To clarify the links between socioeconomic status and the prevalence of ASD (with or without ID) and isolated severe ID | Pop Reg  Cros Sec | childhood disability registry of the administrative area of Haute-Garonne, SW France | Being born 1995 to 2004, ID comorbidity | France | 500 ASD children and 245 children with severe ID (IQ <50) | 500 | age 8 | 80% | ICD-10 by physician | ICD-10 and standardized intelligence tests, mainly WISC | NA | **51%** (256 of 500 ASD) |  |
| Bourke, et al. (2016) | To investigate the prevalence of ID and/or ASD in Western Australia | Pop Reg | IDEA database | Children born and alive 1983- 2010 with an ID and/ or ASD | Australia | 721,645 | 2307 | NA | NA | DSM- 4 | Full scale IQ< 70 | NA | **70,7%** ID (N:1632) |  |
| Van Naarden Braun, et al. (2015) | To examine the prevalence of ASD, ID, and other disabilities over a 15-20 year time period | Pop Reg | MADDSP database | children with ASD registered between 1996–2010; parent(s) or legal guardian(s) residing within a five county metropolitan Atlanta area | USA | 2307 | **1996**:  156  **2000**:  285  **2002**:  337  **2004**:  401 **2006**:  474  **2008**:  601  **2010**:  752 | 8 | 82.4% | ICD -9  DSM- 4 | Full scale IQ< 70 | NA | **1996**:0,585  **2000**:0,4  **2002**:0,491  **2004**:0,411  **2006**:0,376  **2008**:0,414  **2010**:0,366 |  |
| Bowers, et al. (2015) | To characterize the phenotype of males and females with ASD born  preterm versus those born at term | Pop Reg  Cros Sec | Part of a larger sample of subjects with ASD involved in an assessment of med management | Consecutive individuals, <18 years referred to an academic tertiary care ASD center between July 2004 -April 2012 | USA | 883ASD | 883 (preterm 115, term 768) | Mean: 8.3 (SD  3.8 ) | %82.6 Male | DSM-IV-TR | Clinical interviews and review of neuropsychological testing and school reports when available | NA | **Mild:**  full cohort 68 (7.7) preterm 10 (8.7) term 58 (7.6) **Moderate**: full cohort 55 (6.3) preterm 10 (8.7) term 45 (5.9) **Severe**: full cohort 15 (1.7) preterm 3 (2.6) term 12 (1.6) **Profound:** full cohort 2 (0.2) preterm 0 term 2 (0.3) **Borderline intellectual functioning**: full cohort 37 (4.2) preterm 4 (3.5) term 33 (4.3) **Unspecified**: full cohort 120 (13.6) preterm 23 (20.0) term 97 (12.7) **Unknown**: full cohort 25 (2.8) preterm 2 (1.7) term 23 (3.0) | **Mild:**  Female Preterm: 2 (9.5)  Female Term: 16 (12.1) Male Preterm: 8 (8.5)  Male Term: 42 (6.6)  **Moderate:**  Female Preterm: 1 (4.8)  Female Term: 8 (6.1)  Male Preterm: 9 (9.6)  Male Term: 37 (5.9)  **Severe:**  Female Preterm: 3 (14.3)  Female Term: 4 (3.0)  Male Preterm: 0  Male Term: 8 (1.3)  **Profound:**  Female Preterm: 0  Female Term: 0  Male Preterm: 0  Male Term: 2 (0.3)  **Unspecified:**  Female Preterm: 5 (23.8)  Female Term: 19 (14.4)  Male Preterm: 19 (19.2)  Male Term: 78 (12.3) |
| Gordon-Lipkin, et al. (2018) | To compare the comorbidity of  anxiety and mood disorders in children with ASD, with and without ADHD | Pop Reg  Cros Sec | IAN database | Children ages 6 -17 years with diagnosis of ASD | USA | 3319 | 3319 | 6-17 | 83 % M | professional diagnosis and SCQ (total score ≥12) and SRS | parent report on CAQ | NA | **19.6%** |  |
| Chen, et al. (2017) | To investigate the suicide risk for young adults and adolescent with ASD | cohort | Taiwan’s National Health Insurance data | 12–29 year old individuals with ASD and age&sex-matched controls enrolled between 2001 -  2009 and followed to the end of 2011 | Taiwan | 5618 ASD +22,472 control | 5618 (for the total sample of adolescents and young adults) | 12-17 and 18-29 | %78 (for the total sample of adolescents and young adults) | ICD-9 | ICD-9 | NA | **%42.8** (for the total sample of adolescents and young adults) |  |
| Diallo, et al. (2018) | To estimate the prevalence, comorbidities, and service use of people with ASD | cohort | QICDSS data | All residents up to age 24 from January 1, 1996- March 31, 2015 | Canada | 1464600 | 16940 | 1-17 | NA | ICD-9, ICD-10 | ICD-9 | 1.2% | **6%** |  |

| **Table 2. Population-Based Study Characteristics of Attention Deficit Hyperactivity Disorder Comorbidity in Children with Autism Spectrum Disorder** | | | | | | | | | | | | |
| --- | --- | --- | --- | --- | --- | --- | --- | --- | --- | --- | --- | --- |
| **Author (year)** | **Aim of the study** | **Study Design** | **Dataset** | **Inclusion Criteria** | **Country** | **Total (N)** | **ASD Sample** | | | **Diagnostic tools** | | **Prevalence of comorbid ADHD** |
|  |  |  |  |  |  |  | **n** | **Age** | **Male %** | **ASD** | **ADHD** |  |
| Posserud, et al. (2018) | To investigate co-occuring problems in children with ASD | Pop Reg  Cros Sec | BCS database | Children with ADHD, LD, ODD,emotional problems, tics | Norway | 6237 | 226 | 7-9 | 160/66  (70.79%) | ASSQ≥17 | DSM-IV | **43.8%** |
| Neumeyer, et al. (2019) | To observe prevalence of co-occurring conditions | Pop Reg  Cros Sec | Autism Speaks - ATN data | anxiety, adhd, sleep disorders,feeding, speech, developmental delay | USA | 1221 | 1221 | 6-17 years | 83/17 | ADOS/ADOS-2 and DSM4 | Defined by the clinicians at the initial visit | for 6-17 years old **34.5%** |
| Kamimura-Nishimura, et al. (2017) | To examine trends in healthcare provider, ASD and behavioral comorbidities | Pop Reg  Cros Sec | NAMCS and NHAMCSOutpatient Samples | 2-18 years olds between 1994 and 2009 | USA | 158,488 | 1083 | 2-18 years | 4:1  80% | ICD-9 | ICD-9 | **21%** |
| Chen, et al. (2017) | To investigate the suicide risk for young adults and adolescent with ASD | Cohort | Taiwan’s National Health Insurance data | 12–29 year old individuals with ASD and age&sex-matched controls enrolled between 2001 -  2009 and followed to the end of 2011 | Taiwan | 5618 ASD +22,472 control | 5618 (for the total sample of adolescents and young adults) | 12-17 and 18-29 | %78 (for the total sample of adolescents and young adults) | ICD-9 | ICD-9 | **25.3%**  (for the total sample of adolescents and young adults) |
| Bhat (2020) | To assess the prevalence of  a risk for motor impairment or DCD in children with ASD | Cross  sectional | SPARK data | To have motor impairment and DCD comorbidities | USA | 11814 | 11814 | 5-15 | M=12,991, F=3347  (79.5%) | SCQ- total score ≥12 | DCDQ on SPARK database | **40.8 %** |
| Soke, et al. (2018) | To compare the prevalence of various medical and behavioral co-occurring conditions/ symptoms with ASD | Cross-sectional | ADDM 2010 survey year data | children who living in ADDM areas, 4 or 8-years old during the surveillance year, and have ICD of ASD or other developmental disabilities | USA | 1874 ASD  4-year-olds (n = 783) and 8-year-olds (n = 1091) | 1091 (8 year old) | 4 year olds  and 8 year olds | 81.03%M (8 year old) | **1th phase:**  ICD Codes  **2th phase:**  Clinician review based on DSM-4 TR and DSM-5 | ADDM expert clinicians review children's file | **26.05 %** for 8 year olds |
| Rubenstein, et al. (2018) | To estimate trends in documented co-occuring conditions in ASD children | Pop Reg | ADDM | Children born in 1994, 1998, 2000, 2002, parents live in site specific areas in 2002, 2006, 2008, 2010surveillance years | USA | 6379 | **2002**: 932  **2006**:1429  **2008**: 1666  **2010**: 2352 | 8 | 80.8 % | **1th phase:**  ICD Codes  **2th phase:**  Clinician review based on DSM-4 TR and DSM-5 | ADDM expert clinicians review children's file | 2002: **9.4%**  2006: **13.4%**  2008: **12.9%**  2010: **13.6%** |
| Houghton, et al. (2017) | To investigate the prevalence and length of prescriptions by age, psychiatric comorbidity for ASD | Insurance Data Cohort Study | Truven Health MarketScanVR Commercial Database and the Truven Health MarketScan Multi-State Medicaid Database | At least two claims for ASD with ICD-9 codes, of which at least one had to be recorded in 2014, >3 years old and non-Rett | USA | 46,943 commercially-insured and 46,696 Medicaid-insured subjects | Commercially insured**5–11:** 17,420 **12–17:** 15,086  Medicaid **5–11:**19,527  **12–17:** 12,954 | 5-11 12-17 | 80.47 % M between the ages 5-11  79.48 % M between the ages 12-17 | ICD-9 | ICD-9 | **Commercially Insured**  **Ages 5-11:** 40.46%  **Ages 12-17:** 47.73%  **Medicaid**  **Ages 5-11:** 47.67%  **Ages 12-17:** 51.08% |
| Gordon-Lipkin, et al. (2018) | To compare the comorbidity of  anxiety and mood disorders in children with ASD, with and without ADHD | Pop Reg  Cros Sec | IAN database | Children ages 6 -17 years with diagnosis of ASD | USA | 3319 | 3319 | 6-17 | 83 % M | professional diagnosis and SCQ (total score ≥12) and SRS | parent report on CAQ | **45.3%** |
| Diallo, et al. (2018) | To estimate the prevalence, comorbidities, and service use of people with ASD | cohort | QICDSS data | All residents up to age 24 from January 1, 1996- March 31, 2015 | Canada | 1464600 | 16940 | 1-17 | NA | ICD-9, ICD-10 | ICD-9 | **42.0 %** |
| Bowers, et al. (2015) | To characterize the phenotype of males and females with ASD born  preterm versus those born at term | Pop Reg  Cros Sec | Part of a larger sample of subjects with ASD involved in an assessment of med management | Consecutive individuals, <18 years referred to an academic tertiary care ASD center between July 2004 -April 2012 | USA | 883ASD | 883 (preterm 115, term 768) | Mean: 8.3 (SD  3.8 ) | %82.6 Male | DSM-IV-TR | Clinical interviews and review of neuropsychological testing and school reports when available | **full cohort** 62%  **preterm** 15%  **term** 47%  **preterm%14.9, term%6.6** |
| Alexeef, et al. (2017) | To examine medical conditions diagnosed prior to ASD | Matched case-control study | Data from electronic medical records of KPNC | Children who were KPNC members  born  from January 1, 2000 through December 31, 2009 | USA | 42520 | 3911 | 2-12 | 81.8 % male (3198 male) | ICD- 9 | ICD-9 | **6.39 %** (n=250) |
| Romero, et al. (2016) | To explore psychiatric comorbidities and the severity of symptoms that could be relevant for the phenotype characterization in ASD | Sch Pop Cros Sec | Data recruited from educational institutions in Málaga | Children who meet DSM IV-TR criteria for PDD | Spain | 56,839 | 123 PDD | 5-15 | 82% (10.62 mean age) | DSM-IV-TR and the DSM-5  ICD-10 Checklists | DSM-IV-TR and DSM 5 and ICD-10 Checklists | **% 57.7** ADHD |
| Davignon, et al. (2018) | To determine frequency of  medical and psychiatric conditions in a large population of  individuals with ASD | Electronic medical records cohort | KPNC data from 2013-2015 | 14-25 year old individuals as of January 1, 2014, members of KPNC for at least 9 months in each calendar year from 2013 -2015 | USA | 385051 | 4123 | 14-17 | 4:1 | ICD -9  ADOS | ICD - 9 | **14.6%** |
| Hill, et al. (2015) | To determine prevalence of unhealthy weight in ASD | Pop Reg  Cros Sec | Autism Speaks ATN from 2008-2013 | Children ages 2 to 17 with confirmed diagnosis according to DSM 4 and ADOS | USA and Canada | N= 13897  ASD n=5053 control group n=8844 | 5053 | 2-17 | M:4270  F: 783 | DSM-IV, ADOS, VABS 2 | clinical staff measured-IQ<70 | **20.6%** |

| **Table 3. Population-Based Study Characteristics of Anxiety Disorders Comorbidity in Children with Autism Spectrum Disorder** | | | | | | | | | | | | |
| --- | --- | --- | --- | --- | --- | --- | --- | --- | --- | --- | --- | --- |
| **Author (year)** | **Aim of the study** | **Study Design** | **Dataset** | **Inclusion criteria for the study** | **Country** | **Total (N)** | **ASD Sample** | | | **Diagnostic tools** | | **Prevalence of anxiety** |
|  |  |  |  |  |  |  | n | Age | Male % | ASD | Anxiety |  |
| Alexeef, et al. (2017) | To examine medical conditions diagnosed prior to ASD | Matched case-control study | Data from electronic medical records of KPNC | Children who were KPNC members  born  from January 1, 2000 through December 31, 2009 | USA | 42.520 | 3911 ASD | 2-12 | 81.8 % (N: 3198) | ICD-9 | ICD-9 | **3.20%** |
| Bowers, et al. (2015) | To characterize the phenotype of males and females with ASD born  preterm versus those born at term | Pop Reg  Cros Sec | Part of a larger sample of subjects with ASD involved in an assessment of med management | Consecutive individuals, <18 years referred to an academic tertiary care ASD center between July 2004 -April 2012 | USA | 883ASD | 883 (preterm 115, term 768) | Mean: 8.3 | %82.6 Male | DSM-IV-TR | Clinical interviews and review of neuropsychological testing and school reports when available | **9.5% (preterm), 5.3% (term)** |
| Houghton, et al. (2017) | To investigate the prevalence and length of prescriptions by age, psychiatric comorbidity for ASD | Insurance Data Cohort Study | Truven Health MarketScanVR Commercial Database and the Truven Health MarketScan Multi-State Medicaid Database | At least two claims for ASD with ICD-9 codes, of which at least one had to be recorded in 2014, >3 years old and non-Rett | USA | 46,943 commercially-insured and 46,696 Medicaid-insured subjects | Commercially insured**5–11:** 17,420 **12–17:** 15,086  Medicaid **5–11:**19,527  **12–17:** 12,954 | 5-11 12-17 | 80.47 % M between the ages 5-11  79.48 % M between the ages 12-17 | ICD-9 | ICD-9 | **Commercially Insured Data**  **Ages 5-11:** 18.87%  **Ages 12-17:** 30.49%  **Medicaid Data**  **Ages 5-11:** 11.50%  **Ages 12-17:** 17.73% |
| Chen, et al. (2017) | To investigate the suicide risk for young adults and adolescent with ASD | Cohort | Taiwan’s National Health Insurance data | 12–29 year old individuals with ASD and age&sex-matched controls enrolled between 2001 -  2009 and followed to the end of 2011 | Taiwan | 5618 ASD +22,472 control | 5618 (for the total sample of adolesents and young adults) | 12-17 and 18-29 | %78 (for the total sample of adolescents and young adults) | ICD-9 | ICD-9 | **11.2%** |
| Kamimura-Nishimura, et al. (2017) | To examine trends in healthcare provider, ASD and behavioral comorbidities | Pop Reg  Cros Sec | NAMCS and NHAMCSOutpatient Samples | 2-18 years olds between 1994 and 2009 | USA | 158,484 | 1083 | 2-18years | 4:1  80% | ICD-9 | ICD-9 | **7.4%** |
| Rubenstein, et al. (2018) | To estimate trends in documented co-occuring conditions in ASD children | Pop Reg | ADDM | Children born in 1994, 1998, 2000, 2002, parents live in site specific areas in 2002, 2006, 2008, 2010surveillance years | USA | 6379 | **2002**: 932  **2006**:1429  **2008**: 1666  **2010**: 2352 | 8 | 80.8 % | **1th phase:**  ICD Codes  **2th phase:**  Clinician review based on DSM-4 TR and DSM-5 | ADDM expert clinicians review children's file | 2002:**1.1 %**  2006:**1.9 %**  2008: **3.8 %**  2010: **3.7 %** |
| Neumeyer, et al. (2019) | To observe prevalence of co-occurring conditions | Pop Reg  Cros Sec | Autism Speaks - ATN data | anxiety, adhd, sleep disorders,feeding, speech, developmental delay | USA | 1221 | 1221 | 6-17 years | 83/17 | ADOS/ADOS-2 and DSM4 | Defined by the clinicians at the initial visit | **19.7%** |
| Soke, et al.  (2018) | To compare the prevalence of various medical and behavioral co-occurring conditions/ symptoms with ASD | Cross-sectional | ADDM 2010 survey year data | children who living in ADDM areas, 4 or 8-years old during the surveillance year, and have ICD of ASD or other developmental disabilities | USA | 1874 ASD  4-year-olds (n = 783) and 8-year-olds (n = 1091) | 1091 (8 year old) | 4 year olds  and 8 year olds | 81.03%M (8 year old) | **1th phase:**  ICD Codes  **2th phase:**  Clinician review based on DSM-4 TR and DSM-5 | ADDM expert clinicians review children's file | 4 year old: **4.93%**  8 year old: **11.18%** |
| Romero, M. et al (2016) | To explore psychiatric comorbidities and the severity of symptoms that could be relevant for the phenotype characterization in ASD | Sch Pop Cros Sec | Data recruited from educational institutions in Málaga | Children who meet DSM IV-TR criteria for PDD | Spain | 56,839 | 123 PDD | 5-15 | 82% (10.62 mean age) | DSM-IV-TR and the DSM-5  ICD-10 Checklists | DSM-IV-TR and DSM 5 and ICD-10 Checklists | **31.7 %** |
| Gordon-Lipkin, et al. (2020) | To compare the comorbidity of  anxiety and mood disorders in children with ASD, with and without ADHD | Pop Reg  Cros Sec | IAN database | Children ages 6 -17 years with diagnosis of ASD | USA | 3319 | 3319 | 6-17 | 83 % M | professional diagnosis and SCQ (total score ≥12) and SRS | parent report on CAQ | Ages 6-11: **25.7%** and ages 12-17: **45.3%** |
| Davignon, et al. (2018) | To determine frequency of  medical and psychiatric conditions in a large population of  individuals with ASD | Electronic medical records cohort | KPNC data from 2013-2015 | 14-25 year old individuals as of January 1, 2014, members of KPNC for at least 9 months in each calendar year from 2013 -2015 | USA | 385051 | 4123 | 14-17 | 4:1 | ICD -9  ADOS | ICD - 9 | **14.4%** |
| Hill, et al. (2015) | To determine prevalence of unhealthy weight in ASD | Pop Reg  Cros Sec | Autism Speaks ATN from 2008-2013 | Children ages 2 to 17 with confirmed diagnosis according to DSM 4 and ADOS | USA and Canada | N= 13897  ASD n=5053 control group n=8844 | 5053 | 2-17 | M:4270  F: 783 | DSM-IV, ADOS, VABS 2 | clinical staff measured-IQ<70 | **23.2%** |

| **Table 4. Population-Based Study Characteristics of Disruptive Behaviors Comorbidity in Children with Autism Spectrum Disorder** | | | | | | | | | | | | | |
| --- | --- | --- | --- | --- | --- | --- | --- | --- | --- | --- | --- | --- | --- |
| **Author (year)** | **Aim of the study** | **Study Design** | **Dataset** | **Inclusion Criteria** | **Country** | **Total (N)** | **ASD Sample** | | | **Diagnostic tools** | | **Prevalence of comorbid Disruptive Behavior** | **Included Types of Disruptive Behavior** |
|  |  |  |  |  |  |  | **n** | **Age** | **Male %** | **ASD** | **Disruptive Behavior** |  |  |
| Posserud, et al. (2018) | To investigate co-occuring problems in children with ASD | Pop Reg  Cros Sec | BCS database | Children with ADHD, LD, ODD,emotional problems, tics | Norway | 6237 | 226 | 7-9 | 160/66  (70.79%) | DSM-IV  ASSQ≥17 | DSM-IV | **31.9%** | ODD |
| Neumeyer, et al. (2019) | To observe prevalence of co-occurring conditions | Pop Reg  Cros Sec | Autism Speaks - ATN data | anxiety, adhd, sleep disorders,feeding, speech, developmental delay | USA | 1221 | 1221 | 6-17 years | 83/17 | ADOS/ADOS-2 and DSM4 | Defined by the clinicians at the initial visit | for 6-17 years old **6.1%** | Disruptive behavior disorder |
| Kamimura-Nishimura, et al. (2017) | To examine trends in healthcare provider, ASD and behavioral comorbidities | Pop Reg  Cros Sec | NAMCS and NHAMCSOutpatient Samples | 2-18 years olds between 1994 and 2009 | USA | 158,488 | 1083 | 2-18 years | 4:1  80% | ICD-9 | ICD-9 | **7.1%** | Disruptive behaviors |
| Chen, et al. (2017) | To investigate the suicide risk for young adults and adolescent with ASD | Cohort | Taiwan’s National Health Insurance data | 12–29 year old individuals with ASD and age&sex-matched controls enrolled between 2001 -  2009 and followed to the end of 2011 | Taiwan | 5618 ASD +22,472 control | 5618 (for the total sample of adolescents and young adults) | 12-17 and 18-29 | %78 (for the total sample of adolescents and young adults) | ICD-9 | ICD-9 | **6.6%**  (for the total sample of adolescents and young adults) | Disruptive behavior disorder |
| Soke, et al. .(2018) | To compare the prevalence of various medical and behavioral co-occurring conditions/ symptoms with ASD | Cross-sectional | ADDM 2010 survey year data | children who living in ADDM areas, 4 or 8-years old during the surveillance year, and have ICD of ASD or other developmental disabilities | USA | 1874 ASD  4-year-olds (n = 783) and 8-year-olds (n = 1091) | 1091 (8 year old) | 4 year olds  and 8 year olds | 81.03%M (8 year old) | **1th phase:**  ICD Codes  **2th phase:**  Clinician review based on DSM-4 TR and DSM-5 | ADDM expert clinicians review children's file | **4.03 %** for 8 year olds | ODD |
| Rubenstein, et al. (2018) | To estimate trends in documented co-occuring conditions in ASD children | Pop Reg | ADDM | Children born in 1994, 1998, 2000, 2002, parents live in site specific areas in 2002, 2006, 2008, 2010surveillance years | USA | 6379 | **2002**: 932  **2006**:1429  **2008**: 1666  **2010**: 2352 | 8 | 80.8 % | **1th phase:**  ICD Codes  **2th phase:**  Clinician review based on DSM-4 TR and DSM-5 | ADDM expert clinicians review children's file | 2002: **1.0%**  2006: **1.7%**  2008: **1.4%**  2010: **0.6%** | ODD +CD |
| Houghton, et al. (2017) | To investigate the prevalence and length of prescriptions by age, psychiatric comorbidity for ASD | Insurance Data Cohort Study | Truven Health MarketScanVR Commercial Database and the Truven Health MarketScan Multi-State Medicaid Database | At least two claims for ASD with ICD-9 codes, of which at least one had to be recorded in 2014, >3 years old and non-Rett | USA | 46,943 commercially-insured and 46,696 Medicaid-insured subjects | Commercially insured**5–11:** 17,420 **12–17:** 15,086  Medicaid **5–11:**19,527  **12–17:** 12,954 | 5-11 12-17 | 80.47 % M between the ages 5-11  79.48 % M between the ages 12-17 | ICD-9 | ICD-9 | **Commercially Insured Data**  **Ages 5-11:** 11.97%  **Ages 12-17: 1**4.55%  **Medicaid Data**  **Ages 5-11:** 19.67%  **Ages 12-17:** 23.81% | CD |
| Alexeef, et al. (2017) | To examine medical conditions diagnosed prior to ASD | Matched case-control study | Data from electronic medical records of KPNC | Children who were KPNC members  born  from January 1, 2000 through December 31, 2009 | USA | 42520 | 3911 | 2-12 | 81.8 % male (3198 male) | ICD- 9 | ICD-9 | **5.22 %** | Disruptive, impulse, conduct disorders |
| Davignon, et al. (2018) | To determine frequency of  medical and psychiatric conditions in a large population of  individuals with ASD | Electronic medical records cohort | KPNC data from 2013-2015 | 14-25 year old individuals as of January 1, 2014, members of KPNC for at least 9 months in each calendar year from 2013 -2015 | USA | 385051 | 4123 | 14-17 | 4:1 | ICD -9  ADOS | ICD - 9 | **3.5%** | Disruptive, impulse, and conduct disorders |
| Fombonne, et al. (2016) | To estimate ASD prevalence in Mexico | Sch Pop | The Leon Survey | Children were born between Jan 1st and Dec 31th 2003 residing in Leon | Mexico | 12.116 | 36 | 8 | 80.6 %  4.1/1 | SRS  ADI-R  ADOS | Based on a clinical estimate derived from a review of cognitive data available | **69.4%** | Disruptive behavioral problems |

| **Table 5. Population-Based Study Characteristics of Bipolar Disorder Comorbidity in Children with Autism Spectrum Disorder** | | | | | | | | | | | | |
| --- | --- | --- | --- | --- | --- | --- | --- | --- | --- | --- | --- | --- |
| **Author (year)** | **Aim of the study** | **Study Design** | **Dataset** | **Inclusion criteria for the study** | **Country** | **Total (N)** | **ASD Sample** | | | **Diagnostic tools** | | **Prevalence of Bipolar Disorder** |
|  |  |  |  |  |  |  | n | Age | Male % | ASD | Bipolar |  |
| Bowers, et al. (2015) | To characterize the phenotype of males and females with ASD born  preterm versus those born at term | Pop Reg  Cros Sec | Part of a larger sample of subjects with ASD involved in an assessment of med management | Consecutive individuals, <18 years referred to an academic tertiary care ASD center between July 2004 -April 2012 | USA | 883ASD | 883 (preterm 115, term 768) | Mean: 8.3 (SD  3.8 ) | %82.6 | DSM-IV-TR | Clinical interviews and review of neuropsychological testing and school reports when available | **2.5%** |
| Houghton, et al. (2017) | To investigate the prevalence and length of prescriptions by age, psychiatric comorbidity for ASD | Insurance Data Cohort Study | Truven Health MarketScanVR Commercial Database and the Truven Health MarketScan Multi-State Medicaid Database | At least two claims for ASD with ICD-9 codes, of which at least one had to be recorded in 2014, >3 years old and non-Rett | USA | 46,943 commercially-insured and 46,696 Medicaid-insured subjects | Commercially insured**5–11:** 17,420 **12–17:** 15,086  Medicaid **5–11:**19,527  **12–17:** 12,954 | 5-11 12-17 | 80.47 % between the ages 5-11  79.48 % between the ages 12-17 | ICD-9 | ICD-9 | **Commercially Insured Data**  **Ages 5-11:** 1.43%  **Ages 12-17:** 5.91%  **Medicaid Data**  **Ages 5-11:** 3.93%  **Ages 12-17:** 10.97% |
| Chen, et al. (2017) | To investigate the suicide risk for young adults and adolescent with ASD | Cohort | Taiwan’s National Health Insurance data | 12–29 year old individuals with ASD and age&sex-matched controls enrolled between 2001 -  2009 and followed to the end of 2011 | Taiwan | 5618 ASD +22,472 control | 5618 (for the total sample of adolescents and young adults) | 12-17 and 18-29 | %78 (for the total sample of adolescents and young adults) | ICD-9 | ICD-9 | **8.4%** |
| Rubenstein, et al. (2018) | To estimate trends in documented co-occuring conditions in ASD children | Pop Reg | ADDM | Children born in 1994, 1998, 2000, 2002, parents live in site specific areas in 2002, 2006, 2008, 2010surveillance years | USA | 6379 | **2002**: 932  **2006**:1429  **2008**: 1666  **2010**: 2352 | 8 | 80.8 % | **1th phase:**  ICD Codes  **2th phase:**  Clinician review based on DSM-4 TR and DSM-5 | ADDM expert clinicians review children's file | 2002:**0.2 %**  2006:**0.6 %**  2008: **0.5 %**  2010: **0.3 %** |
| Davignon, et al. (2018) | To determine frequency of  medical and psychiatric conditions in a large population of  individuals with ASD | Electronic medical records cohort | KPNC data from 2013-2015 | 14-25 year old individuals as of January 1, 2014, members of KPNC for at least 9 months in each calendar year from 2013 -2015 | USA | 385051 | 4123 | 14-17 | 4:1 | ICD -9  ADOS | ICD - 9 | **6.4%** |
| Selten, et al. (2015) | To determine the risk of non-affective psychosis and bipolar in individuals with ASD | case-control study | Stockholm Youth Cohort | children 17 year old or younger with ASD | Sweden | 735096 | 9062 | 0-17  (mean age 15.3) | 73% | DSM-IV, ICD-10 | ICD-10 | **0,6%** in ASD group  and **0.1%** for control group |

| **Table 6. Population-Based Study Characteristics of Depression Comorbidity in Children with Autism Spectrum Disorder** | | | | | | | | | | | | |
| --- | --- | --- | --- | --- | --- | --- | --- | --- | --- | --- | --- | --- |
| **Author (year)** | **Aim of the study** | **Study Design** | **Dataset** | **Inclusion criteria for the study** | **Country** | **Total (N)** | **ASD Sample** | | | **Diagnostic tools** | | **Prevalence of Depression** |
|  |  |  |  |  |  |  | n | Age | Male % | ASD | Depression |  |
| Bowers, et al. (2015) | To characterize the phenotype of males and females with ASD born  preterm versus those born at term | Pop Reg  Cros Sec | Part of a larger sample of subjects with ASD involved in an assessment of med management | Consecutive individuals, <18 years referred to an academic tertiary care ASD center between July 2004 -April 2012 | USA | 883ASD | 883 (preterm 115, term 768) | Mean: 8.3 (SD  3.8 ) | %82.6 | DSM-IV-TR | Clinical interviews and review of neuropsychological testing and school reports when available | **3.2%** |
| Houghton, et al. (2017) | To investigate the prevalence and length of prescriptions by age, psychiatric comorbidity for ASD | Insurance Data Cohort Study | Truven Health MarketScanVR Commercial Database and the Truven Health MarketScan Multi-State Medicaid Database | At least two claims for ASD with ICD-9 codes, of which at least one had to be recorded in 2014, >3 years old and non-Rett | USA | 46,943 commercially-insured and 46,696 Medicaid-insured subjects | Commercially insured**5–11:** 17,420 **12–17:** 15,086  Medicaid **5–11:**19,527  **12–17:** 12,954 | 5-11 12-17 | 80.47 % between the ages 5-11  79.48 % between the ages 12-17 | ICD-9 | ICD-9 | **Commercially Insured**  **Ages 5-11:** 2.42%  **Ages 12-17:** 13.11%  **Medicaid**  **Ages 5-11:** 5.08%  **Ages 12-17:** 12.31% |
| Chen, et al. (2017) | To investigate the suicide risk for young adults and adolescent with ASD | Cohort | Taiwan’s National Health Insurance data | 12–29 year old individuals with ASD and age&sex-matched controls enrolled between 2001 -  2009 and followed to the end of 2011 | Taiwan | 5618 ASD +22,472 control | 5618 (for the total sample of adolescents and young adults) | 12-17 and 18-29 | %78 (for the total sample of adolescents and young adults) | ICD-9 | ICD-9 | **16%** |
| Rubenstein, et al. (2018) | To estimate trends in documented co-occuring conditions in ASD children | Pop Reg | ADDM | Children born in 1994, 1998, 2000, 2002, parents live in site specific areas in 2002, 2006, 2008, 2010surveillance years | USA | 6379 | **2002**: 932  **2006**:1429  **2008**: 1666  **2010**: 2352 | 8 | 80.8 % | **1th phase:**  ICD Codes  **2th phase:**  Clinician review based on DSM-4 TR and DSM-5 | ADDM expert clinicians review children's file | 2002:**0.1 %**  2006:**0.5 %**  2008: **0.1 %**  2010: **0.3 %** |
| Davignon, et al. (2018) | To determine frequency of  medical and psychiatric conditions in a large population of  individuals with ASD | Electronic medical records cohort | KPNC data from 2013-2015 | 14-25 year old individuals as of January 1, 2014, members of KPNC for at least 9 months in each calendar year from 2013 -2015 | USA | 385051 | 4123 | 14-17 | 4:1 | ICD -9  ADOS | ICD - 9 | **9.9%** |
| Alexeeff, et al. (2017) | To examine medical conditions diagnosed prior to ASD | Matched case-control study | Data from electronic medical records of KPNC | Children who were KPNC members  born  from January 1, 2000 through December 31, 2009 | USA | 42520 | 3911 | 2-12 | 81.8 % male (3198 male) | ICD- 9 | ICD-9 | **0.59%** |

| **Table 7. Population-Based Study Characteristics of Obsessive-Compulsive Disorder Comorbidity in Children with Autism Spectrum Disorder** | | | | | | | | | | | | |
| --- | --- | --- | --- | --- | --- | --- | --- | --- | --- | --- | --- | --- |
| **Author (year)** | **Aim of the study** | **Study Design** | **Dataset** | **Inclusion criteria for the study** | **Country** | **Total (N)** | **ASD Sample** | | | **Diagnostic tools** | | **Prevalence of OCD** |
|  |  |  |  |  |  |  | n | Age | Male % | ASD | OCD |  |
| Bowers, et al. (2015) | To characterize the phenotype of males and females with ASD born  preterm versus those born at term | Pop Reg  Cros Sec | Part of a larger sample of subjects with ASD involved in an assessment of med management | Consecutive individuals, <18 years referred to an academic tertiary care ASD center between July 2004 -April 2012 | USA | 883ASD | 883 (preterm 115, term 768) | Mean: 8.3 | %82.6 Male | DSM-IV-TR | Clinical interviews and review of neuropsychological testing and school reports when available | **1.9%** |
| Rubenstein, et al. (2018) | To estimate trends in documented co-occuring conditions in ASD children | Pop Reg | ADDM | Children born in 1994, 1998, 2000, 2002, parents live in site specific areas in 2002, 2006, 2008, 2010surveillance years | USA | 6379 | **2002**: 932  **2006**:1429  **2008**: 1666  **2010**: 2352 | 8 | 80.8 % | **1th phase:**  ICD Codes  **2th phase:**  Clinician review based on DSM-4 TR and DSM-5 | ADDM expert clinicians review children's file | 2002:**0.1 %**  2006:**0.3 %**  2008: **0.2 %**  2010: **0.4 %** |
| Davignon, et al. (2018) | To determine frequency of  medical and psychiatric conditions in a large population of  individuals with ASD | Electronic medical records cohort | KPNC data from 2013-2015 | 14-25 year old individuals as of January 1, 2014, members of KPNC for at least 9 months in each calendar year from 2013 -2015 | USA | 385051 | 4123 | 14-17 | 4:1 | ICD -9  ADOS | ICD - 9 | **2.3%** |
| Posserud, et al. (2018) | To investigate co-occuring problems in children with ASD | Pop Reg  Cros Sec | BCS database | Children with ADHD, LD, ODD,emotional problems, tics | Norway | 6237 | 226 | 7-9 | 160/66  (70.79%) | ASSQ≥17 | DSM-IV | **47.3%** |
| Romero, et al. (2016) | To explore psychiatric comorbidities and the severity of symptoms that could be relevant for the phenotype characterization in ASD | Sch Pop Cros Sec | Data recruited from educational institutions in Málaga | Children who meet DSM IV-TR criteria for PDD | Spain | 56,839 | 123 PDD | 5-15 | 82% (10.62 mean age) | DSM-IV-TR and the DSM-5  ICD-10 Checklists | DSM-IV-TR and DSM 5 and ICD-10 Checklists | **40.7%** |

| **Table 8. Population-Based Study Characteristics of Psychosis Comorbidity in Children with Autism Spectrum Disorder** | | | | | | | | | | | | |
| --- | --- | --- | --- | --- | --- | --- | --- | --- | --- | --- | --- | --- |
| **Author (year)** | **Aim of the study** | **Study Design** | **Dataset** | **Inclusion criteria for the study** | **Country** | **Total (N)** | **ASD Sample** | | | **Diagnostic tools** | | **Prevalence of Psychosis** |
|  |  |  |  |  |  |  | n | Age | Male % | ASD | Psychosis |  |
| Bowers, et al. (2015) | To characterize the phenotype of males and females with ASD born  preterm versus those born at term | Pop Reg  Cros Sec | Part of a larger sample of subjects with ASD involved in an assessment of med management | Consecutive individuals, <18 years referred to an academic tertiary care ASD center between July 2004 -April 2012 | USA | 883ASD | 883 (preterm 115, term 768) | Mean: 8.3 | %82.6 | DSM-IV-TR | Clinical interviews and review of neuropsychological testing and school reports when available | **0.3% (n=3)** full cohort  **0** preterm**, 0.4%** term |
| Houghton, et al. (2017) | To investigate the prevalence and length of prescriptions by age, psychiatric comorbidity for ASD | Insurance Data Cohort Study | Truven Health MarketScanVR Commercial Database and the Truven Health MarketScan Multi-State Medicaid Database | At least two claims for ASD with ICD-9 codes, of which at least one had to be recorded in 2014, >3 years old and non-Rett | USA | 46,943 commercially-insured and 46,696 Medicaid-insured subjects | Commercially insured**5–11:** 17,420 **12–17:** 15,086  Medicaid **5–11:**19,527  **12–17:** 12,954 | 5-11 12-17 | 80.47 % between the ages 5-11  79.48 % between the ages 12-17 | ICD-9 | ICD-9 | **Commercially Insured**  **Ages 5-11:** 0.12%  **Ages 12-17:** 0.75%  **Medicaid**  **Ages 5-11:** 0.30%  **Ages 12-17:** 1.64% |
| Davignon, et al. (2018) | To determine frequency of  medical and psychiatric conditions in a large population of  individuals with ASD | Electronic medical records cohort | KPNC data from 2013-2015 | 14-25 year old individuals as of January 1, 2014, members of KPNC for at least 9 months in each calendar year from 2013 -2015 | USA | 385051 | 4123 | 14-17 | 4:1 | ICD -9  ADOS | ICD - 9 | psychoses: **1.7%** |
| Selten, et al. (2015) | To determine the risk of non-affective psychosis and bipolar in individuals with ASD | case-control study | Stockholm Youth Cohort | children 17 year old or younger with ASD and Bipolar comorbidity | Sweden | 735096 | 9062 | 0-17  (mean age 15.3) | 73% | DSM-IV, ICD-10 | ICD-10 | nonaffective psychotic disorder: **0.6 %**  schizophrenia: **0.1 %** |

| **Table 9. Population-Based Study Characteristics of Sleep Disorders Comorbidity in Children with Autism Spectrum Disorder** | | | | | | | | | | | | |
| --- | --- | --- | --- | --- | --- | --- | --- | --- | --- | --- | --- | --- |
| **Author (year)** | **Aim of the study** | **Study Design** | **Dataset** | **Inclusion criteria for the study** | **Country** | **Total (N)** | **Sample** | | | **Diagnostic tools** | | **Prevalence of Sleep Disorders** |
|  |  |  |  |  |  |  | n | Age | Male % | ASD | Sleep Disorder |  |
| Aldinger, et al. (2015) | To research co-occurring medical problems in ASD patients | Pop Reg | AGRE and SSC databases | detailed medical information included families | USA | 728+627+2623(multiplex complex, cluster analysis, SSC) | 3978 | mean age : multiplex9.2  cluster 9.3  SSC 9 | multiplex 78.3%  cluster 79.7%  SSC 86.7% | ADI-R  SRS | parent report | multiplex **55.5%**  cluster **55%**  SSC **72.5%** |
| Alexeeff, et al. (2017) | To examine medical conditions diagnosed prior to ASD | Matched case-control study | Data from electronic medical records of KPNC | Children who were KPNC members  born  from January 1, 2000 through December 31, 2009 | USA | 42520 | 3911 | 2-12 | 81.8 % male (3198 male) | ICD- 9 | ICD-9 | **%4.83** dyssomnia |
| Houghton, et al. (2017) | To investigate the prevalence and length of prescriptions by age, psychiatric comorbidity for ASD | Insurance Data Cohort Study | Truven Health MarketScanVR Commercial Database and the Truven Health MarketScan Multi-State Medicaid Database | At least two claims for ASD with ICD-9 codes, of which at least one had to be recorded in 2014, >3 years old and non-Rett | USA | 46,943 commercially-insured and 46,696 Medicaid-insured subjects | **Commercially insured** **5–11:** 17,420 **12–17:** 15,086  **Medicaid 5–11:**19,527  **12–17:** 12,954 | 5-11 12-17 | 80.47 % M between the ages 5-11  79.48 % M between the ages 12-17 | ICD-9 | ICD-9 | **Commercially Insured**  **Ages 5-11:** 5.87%  **Ages 12-17:** 5.14%  **Medicaid**  **Ages 5-11:** 10.22%  **Ages 12-17:** 8.85% |
| Ezell, et al.  (2016) | To examine the patterns of Diagnosis and Co-occurring Symptoms in Adopted Children With ASD | Pop Reg  Cros Sec | ATN Data | Individuals who had gender, age at enrollment, and ethnicity information available and excluded those with an ASD residual state diagnosis | USA | 5787 ASD  (non-adopted 5624, adopted 163) | 5787 ASD | 1.5-17.6 | 83.8% | DSM-IV  ADOS | CSHQ | adopted group **42.9%**  nonadopted **%30.7** |
| Elrod, et al.  (2016) | To determine prevalence of diagnosed sleep disorder on ASD | Pop Reg Retro | MHS database | children with ASD aged 2 to 18 years enrolled in MHS from 2000 to 2013 | USA | 48762 ASD | 48762 ASD and control: (243,810) | 2-18 years | 80% | ICD-9 | ICD-9 | **30.66%** |
| Soke, et al.  (2018) | To compare the prevalence of various medical and behavioral co-occurring conditions/ symptoms with ASD | Cross-sectional | ADDM 2010 survey year data | children who living in ADDM areas, 4 or 8-years old during the surveillance year, and have ICD of ASD or other developmental disabilities | USA | 1874 ASD  4-year-olds (n = 783) and 8-year-olds (n = 1091) | 1091 (8 year old) | 4 year olds  and 8 year olds | 81.03%M (8 year old) | **1th phase:**  ICD Codes  **2th phase:**  Clinician review based on DSM-4 TR and DSM-5 | ADDM expert clinicians review children's file | 4 year old: **26.95%**  8 year old: **37.12%** |
| Neumeyer, et al. (2019) | To observe prevalence of co-occurring conditions | Pop Reg  Cros Sec | Autism Speaks - ATN data | anxiety, adhd, sleep disorders,feeding, speech, developmental delay | USA | 1221 | 1221 | 6-17 years | 83/17 | ADOS/ADOS-2 and DSM4 | Defined by the clinicians at the initial visit | **24.3%** |
| Davignon, et al. (2018) | To determine frequency of  medical and psychiatric conditions in a large population of  individuals with ASD | Electronic medical records cohort | KPNC data from 2013-2015 | 14-25 year old individuals as of January 1, 2014, members of KPNC for at least 9 months in each calendar year from 2013 -2015 | USA | 385051 | 4123 | 14-17 | 4:1 | ICD -9  ADOS | ICD - 9 | **6.2%** |

| **Table 10. Population-Based Study Characteristics of Other (Sucide, Bullying, Trauma, Tic Disorder, Gender Identity Disorder and Enuresis/Encopresis) Psychiatric Comorbidities in Children with Autism Spectrum Disorder** | | | | | | | | | | | | |
| --- | --- | --- | --- | --- | --- | --- | --- | --- | --- | --- | --- | --- |
| **Author (year)** | **Aim of the study** | **Study Design** | **Dataset** | **Inclusion criteria for the study** | **Country** | **N (total)** | **ASD Sample** | | | **Diagnostic tools** | | **Prevalence of Other Comorbidities** |
|  |  |  |  |  |  |  | n | Age | Male % | ASD | Comorbid Disorder |  |
| Chen, et al. (2017) | To investigate the suicide risk for young adults and adolescent with ASD | Cohort | Taiwan’s National Health Insurance data | 12–29 year old individuals with ASD and age&sex-matched controls enrolled between 2001 -  2009 and followed to the end of 2011 | Taiwan | 5618 ASD +22,472 control | 5618 (for the total sample of adolescents and young adults) | 12-17 and 18-29 | %78 (for the total sample of adolescents and young adults) | ICD-9 | ICD-9 | suicide attempts **3.9%** (for the total sample of adolescents and young adults) |
| Culpin, et al. (2018) | To examine the relationship of ASD diagnosis and suicidal thoughts, plans, self-harm and depression | Pop Reg | ALSPAC | delivery dates between April 1, 1991, and December 31, 1992 who are alive at the age of 7 | UK | 14,684 | 166 ASD and 1151 in high risk ASD group | 16 year old | %80 male | ICD-10, ALSPAC questionnaire | self harm questions based on CASE study and additionally Short Moods and Feelings Questionnaire | Suicidal thoughts: **13.9%**  Suicidal plans: **4.5%**  Self-harm without suicidal intent: **11.9%**  Self-harming with suicidal intent: **6.9%** |
| Kirby, et al. (2019) | To determine incidence of suicide among individuals with ASD | Pop Reg  Retro | Four sources: URADD, UPDB, IBIS‐PH datas, and statewide suicide surveillance data collected by the Utah OME | individuals registered between 1998- 2017, observed in 5‐year intervals for 20 years | USA | 28824 | 28824 | 5-85 (stratified only 5-30) | 76% male | ICD-9 | inclusion in the OME suicide data- set. | Death by suicide:  **0.17%** |
| Hwang, et al. (2018) | To determine relationship between ASD and bullying | Sch Pop Cros Sec | Elementary school children in a large suburb of Seoul, South Korea, between September 2005 and August 2009. | families of 16 schools that agreed to participate | South Korea | 22,382 | 169 ASD, 86 of them with confirmed diagnosis | 7-12 year | %80 Male | DSM-V, BASC-2-PRS, ASSQ | bullying experience from BASC-2-PRS | parental report of child being victim of bullying **19.2%** |
| Chan, et al (2018) | To determine child victimization and disability | Sch Pop Cros Sec | Primary or secondary education students (around 6–18 years of age), in Hong Kong in 2016-2017 | Children with disabilities | Hong Kong | 4114 | 330 | 6-18  (12.32 mean age) | %62.9 for children with disabilities | Asking whether the child had ever received diagnosis of a specific disorder or problem and integrating WHO category | Child victimization questionnaire | bullying **21%**  cyber bullying **22%**  Peer and sibling victimization **21.5%** |
| Rose CA, et al (2015) | To assess prevalence of bullying on youth with disabilities | Sch Pop Cros Sec | Participants from17 middle schools | Students in Grades 6 to 12 | USA | 14.508 | 66 | Mean age 13.8 | NA for ASD | IDEA diagnosis criteria | University of Illinois Victimization Scale, Online Harassment Victimization, Relational Aggression Victimization, University of Illinois Bully Scale, University of Illinois Fight Scale, Relational Aggression Perpetration | bullying **19.4%**  fighting **19.4%**  relational aggression **18.5%** |
| Bowers, et al (2015) | To characterize the phenotype of males and females with ASD born  preterm versus those born at term | Pop Reg  Cros Sec | Part of a larger sample of subjects with ASD involved in an assessment of med management | Consecutive individuals, <18 years referred to an academic tertiary care ASD center between July 2004 -April 2012 | USA | 883ASD | 883 (preterm 115, term 768) | Mean: 8.3 (SD  3.8 ) | %82.6 Male | DSM-IV-TR | Clinical interviews and review of neuropsychological testing and school reports when available | Tic disorder  **3.3%** full cohort  **2.6%** preterm**, 3.4%** term |
| Posserud, et al (2016) | To investigate co-occuring problems in children with ASD | Pop Reg  Cros Sec | BCS database | Children with ADHD, LD, ODD,emotional problems, tics | Norway | 6237 | 226 | 7-9 | 160/66  (70.79%) | DSM-IV, ASSQ≥17 | BCS questionnaire, SDQ, DSM-IV | Tics **73.9%** |
| Berg, et al. (2016) | To identify the prevalence of ACEs among families of children with and without ASD | Pop Reg  Cros Sec | 2011 to 2012 National Survey of Children's Health data | parents of children age 3 to 17 years in 2011 to 2012 | USA | 61 652 | 1611  (mild ASD=879 or moderate/severe ASD=723) | 3-17  (mean 10.4) | 82% | Parents reports on a health care provider ever telling their child had ‘ASD, PDD, or other ASD’ and whether these diagnoses were currently applicable | A modified version of the CDC–Kaiser ACE scale | **50.9%** exposed to at least one ACE  **10.2%** experiencing between four and nine ACEs  income insufficiency **39.7%**  neighborhood violence **11.4%**  parental divorce **27.9%**  mental illness **18.2%**  substance abuse **13.5%** |
| Hoch & Youssef, (2020) | To examine the relationship of exposure to PTEs and trauma related diagnoses  in ASD and DD | Cros Sec | A large sample from mental health provider - Fraser | registered between August of 2013 and February of 2018 | USA | 7695 | NA | NA | 77.4%  in ASD group | ICAD-9, ICD-10, DSM-IV | CBCL, VABS, SDQ, interview for trauma | **23.5%**  reported trauma  **10.2%** living in two or more living situations  **75.4%** at least one negative life event **4.25%** of trauma specific diagnosis |
| Miesen, et al (2018) | To determine the prevalence of gender dysphoria in adolescents with ASD | Cros Sec | a from LKH,a tertiary mental health clinic specialized in the treatment of ASD | individuals referred to LKH between March 2010 and October 2014 for treatment of ASD and control group from YSR | Netherlands | 1589 adolescents | 573 | *Mean* age = 15.98 | %81.8 Male (469/573) | Clinical diagnosis, CSBQ, DSM | YSR questionnaire | **6.5%** self reported wish to be of the opposite gender |
| Alabaf, et al. (2019) | To identify the prevalence of defined physical problems in a nationwide population of twins with and without NDDs | Pop Reg | Data of CATSS -ongoing | all twins born between the 31st of June 1992 through the 31st of December 2006 | Sweden | 28,058 children, all twins | 1021 (NDD 91 ASD, 108 ASD+ADHD, 50 ASD+LD, 52 ASD+ADHD+LD) | 9-year-olds    and  12-year-olds | 66% (N:672) | A-TAC interview based on the diagnostic criteria of DSM-IV | A-TAC interview based on the diagnostic criteria of DSM-IV | daytime enuresis: **7.4%**, encopresis:**4.0%** |
